# Arterial Medial Calcification through Enhanced small Extracellular Vesicle Release in Smooth Muscle-Specific *Asah1* Gene Knockout Mice

**DOI:** 10.1038/s41598-020-58568-5

**Published:** 2020-02-03

**Authors:** Owais M. Bhat, Guangbi Li, Xinxu Yuan, Dandan Huang, Erich Gulbins, Rakesh C. Kukreja, Pin-Lan Li

**Affiliations:** 1 Department of Pharmacology and Toxicology, Virginia Commonwealth University, School of Medicine, Richmond, VA 23298 USA; 2 Department of Molecular Biology, University of Duisburg-Essen, Essen, Germany and Dept. of Surgery, University of Cincinnati, Cincinnati, USA; 3 VCU Pauley Heart Center, Division of Cardiology, Virginia Commonwealth University, 1101 East Marshall Street, Richmond, VA 23298-0204 USA

**Keywords:** Cellular imaging, Immunochemistry

## Abstract

Arterial medial calcification (AMC) involves an increased small extracellular vesicle (sEV) secretion and apatite calcium precipitation in the arterial wall. The mechanisms mediating AMC remain poorly understood. In the present study, smooth muscle-specific acid ceramidase (Ac) gene knockout mice (*Asah1*^fl/fl^/SM^Cre^) were used to demonstrate the role of lysosomal ceramide signaling pathway in AMC. *Asah1*^fl/fl^/SM^Cre^ mice were found to have more severe AMC in both aorta and coronary arteries compared to their littermates (*Asah1*^fl/fl^/SM^wt^ and WT/WT mice) after receiving a high dose vitamin D. These mice also had pronounced upregulation of osteopontin and RUNX2 (osteogenic markers), CD63, AnX2 (sEV markers) and ALP expression (mineralization marker) in the arterial media. In cultured coronary arterial smooth muscle cells (CASMCs) from *Asah1*^fl/fl^/SM^Cre^ mice, high dose of P_i_ led to a significantly increased calcium deposition, phenotypic change and sEV secretion compared to WT CASMCs, which was associated with reduced lysosome-multivesicular body (MVB) interaction. Also, GW4869, sEV release inhibitor decreased sEV secretion and calcification in these cells. Lysosomal transient receptor potential mucolipin 1 (TRPML1) channels regulating lysosome interaction with MVBs were found remarkably inhibited in *Asah1*^fl/fl^/SM^Cre^ CASMCs as shown by GCaMP3 Ca^2+^ imaging and Port-a-Patch patch clamping of lysosomes. Lysosomal Ac in SMCs controls sEV release by regulating lysosomal TRPML1 channel activity and lysosome-MVB interaction, which importantly contributes to phenotypic transition and AMC.

## Introduction

Arterial calcification involves the accumulation or deposition of apatite calcium salts within the vascular wall that has been associated with aging, atherosclerosis, diabetes mellitus and chronic kidney disease (CKD)^1^. Anatomically, arterial calcification is classified into intimal and medial calcification^2^. The intimal calcification often causes occlusion of the arteries, which is characterized by accumulation of lipids, inflammation, and fibrosis observed as irregular scattered deposits in the atherosclerotic plaques^3^. The arterial medial calcification (AMC) increases arterial stiffness, which is detected as continuous linear hydroxyapatite deposits in the absence of inflammatory cells along the internal elastic lamina. It is frequently observed in the elderly people or in patients with diabetic mellitus and chronic renal failure^4,5^. Literature cites that under normal physiological conditions, spontaneous accumulation of calcium and phosphate levels doesn’t induce arterial calcification because minerals (Ca/P_i_) are tightly balanced in the vasculature^6,7^. Increased intracellular phosphate levels in vascular smooth muscle cells (VSMCs) directly drive their osteogenic differentiation and mineralization, inducing expression of osteogenic markers like RUNX2 and osteopontin^8,9^, associated with secretion of matrix vesicles as primary nucleation sites for calcification^7,9^. Several studies including *ex vivo* human samples and animal models of arterial calcification reported that free serum Ca/P_i_ levels resulted in ossification of soft tissue^10–12^. Human studies have shown that increased serum Ca/P_i_ levels are correlated with the development and progression of calcification^13^. Hence, this mineral imbalance actively stimulate phenotypic transformation of VSMCs during calcification. High doses of vitamin D (Vit D) leads to AMC in mouse models via VSMCs RUNX2 expression^14^, likewise in CKD patients high doses of Vit D is correlated with severity of calcification^15^. Animal studies have shown that physiologically Vit D promotes AMC through abnormal mineral metabolism (Ca/P_i_), which as reported lead to vascular osteogenesis and mineralization^16,17^. However, more studies are required to explain the clear mechanisms of action by which excess exogenous Vit D promotes AMC under *in vivo* conditions.

It has been reported that a common mechanistic pathway that can regulate the arterial medial calcification involves the substantial increase in extracellular vesicles (EVs) in the vascular interstitial space, especially, the small extracellular vesicles (sEVs) or exosomes (40–100 or to 140 nm in size). Such sEVs are in particular produced and released from arterial SMCs^18–20^. Although, the mechanisms mediating sEV release and consequent AMC is still unknown. There are numerous studies which demonstrated that extracellular vesicles (EVs) originate from different subcellular membrane compartments and are released into the interstitial space, regulator of cell-to-cell communications or signaling. Different from other EVs, sEV/exosomes are formed through the endocytic process and released from intracellular multivesicular bodies (MVBs) through an active process. EVs or exosomes have been extensively studied for their biogenesis and related function in cell-to-cell communication and in the pathogenesis of different diseases including cardiovascular diseases^21,22^. In human VSMCs, recent studies revealed that exosomes are originated from a subset of late endosomal compartment, MVBs^18^. Like matrix vesicles (MVs) from bone cells, exosomes from mineralized SMCs are characterized as small electron dense spherical nanoparticles (50–200 nm) composed of calcium and phosphorus, alkaline phosphatase (ALP), and the membrane proteins annexins^23^. Recent studies have indicated that sphingolipid-mediated signaling plays a crucial role in the regulation of MVs secretion and vascular calcification. Sphingomyelin phosphodiesterase 3 (SMPD3, neutral sphingomyelinase) activation and cytoskeletal rearrangements in synthetic VSMCs led to MVB trafficking and elevated exosome secretion^18^, and ceramide (CER) derived from SMPD3 triggers budding of sEV into multivesicular endosomes^24^. In this regard, lysosome-mediated autophagic flux has been reported to determine the fate of MVBs, thereby controlling the release of sEVs^25^. In human arterial SMCs, 7-ketocholesterol (7-KC)-induced oxidative stress caused deficiency of autophagosome and lysosome fusion, which promotes vascular calcification^26^. Dai *et al*. have reported that P_i_-induced VSMC calcification inhibits autophagosome formation^27^. Mutations of VPS4 gene required for MVB maturation and fusion with lysosome increased the levels of EV-associated proteins within cells^28^. This active and fine-controlled lysosome function has also been shown by other studies including ours in coronary arterial smooth muscle cells (CASMCs)^29–31^. It is now imperative to investigate how this lysosome regulation works to lead to sEV secretion from SMCs resulting in AMC.

Lysosome trafficking is an important process that controls the interaction of lysosomes with autophagosomes or MVBs. Recent studies have reported that lysosomal TRPML1 channel activity regulated by sphingolipids is a key mechanism determining lysosome trafficking. In this regard, accumulated sphingomyelin inhibited its activity and reduced lysosomal Ca^2+^ release, leading to failure of lysosome trafficking and lysosomal storage disease as shown in Niemann-Pick disease^32,33^. It remains unknown whether this lysosome TRPML1 channel-mediated Ca^2+^ release is involved in lysosome-MVB interaction and how this Ca^2+^ signaling pathway is regulating sphingolipids associated with sEV release. In the present study, we hypothesized that acid ceramidase (Ac)-mediated sphingolipid signaling may gate TRPML1 channel activity in SMCs thereby regulating lysosome fusion to MVBs that govern sEV excretion. We first examined whether SM-specific deletion of Ac gene, namely *Asah1*, enhances the osteogenic differentiation, sEV secretion, and mineral deposition in the arterial wall of a mouse model of AMC. We further investigated whether this Ac deletion indeed results in osteogenesis via enhanced sEV secretion and lysosome-MVB interaction in primary cultures of CASMCs. Finally, we determined whether Ac-mediated lysosome interactions with MVBs are due to its regulatory action on lysosomal TRPML1 channel-mediated Ca^2+^ signaling. Our results demonstrate that Ac-associated sphingolipids importantly controls TRPML1 channel activity and regulates lysosome-MVB interaction, leading prolongation of MVB fate and consequent enhancement of sEV release. This enhanced sEV release plays a crucial role in the SMC phenotype transition to osteogenic and the development of AMC.

## Results

### Characterization of *Asah1*^fl/fl^/SM^Cre^ mice

*Asah1*^fl/fl^/SM^Cre^ mice were generated by crossing the *Asah1* floxed mice (*Asah1*^fl/fl^)^34^ with SM22α-Cre transgenic mice (SM^Cre^ mice from the Jackson laboratory, B6.129S6-*Tagln*^*tm2(cre)Yec*^/J, Stock No: 006878 | SM22α-creKI). Using mice from the F1 generation with heterozygous Cre expression, namely, *Asah1*^fl/fl^/SM^Cre^, a backcross with the *Asah1*^fl/fl^ mice to generate *Asah1*^fl/fl^/SM^Cre^ mice containing the Cre transgene in SMCs. Deletion of the *Asah1* and transgene of Cre were verified by PCR analysis. As shown in Supplementary Fig. S1A, *Asah1*^fl/fl^/SM^Cre^ has 2 positive PCR products including 758 bp for Cre and 585 bp for floxed *Asah1* gene. *Asah1*^fl/fl^/SM^wt^ mice had positive floxed *Asah1* gene (585 bp), but no Cre (758 bp). WT/WT (*Asah1*^wt^/SM^wt^) mice only had wild type *Asah1* gene (482 bp), but not floxed *Asah1* and Cre gene. Cre-mediated SM-specific recombination was also validated by breeding the *Asah1*^fl/fl^/SM^Cre^ mice with lacZ/EGFP (ZEG) double reporter mice to produce an *Asah1*^fl/fl^/SM^Cre^/ZEG strain. ZEG mice carry a floxed blocker of the reporter gene, enhanced green fluorescent protein (EGFP). Cre excision of the blocker in *Asah1*^fl/fl^/SM^Cre^/ZEG mice activated EGFP to produce green fluorescence as detected by *in vivo* imaging in mouse and in the dissected heart and aorta (Supplementary Fig. S1B). In addition, ZEG mice also carry a floxed lacZ gene with CMV promoter for continuous expression of β-galactosidase (lacZ product). Cre excision of LacZ gene in *Asah1*^fl/fl^/SM^Cre^/ZEG mice led to negative β-gal staining in the coronary arterial wall as shown in Supplementary Fig. S1C (loss of blue staining). Using confocal microscopy, Ac protein was not detected in arterial SMCs and their lysosomes of *Asah1*^fl/fl^/SM^Cre^ mice, as shown by the Ac of its co-localization with α-SMA as SMC marker or lysosomal associated membrane protein-1 (Lamp-1) as a lysosome marker. Ac was detected in arterial SMCs of WT/WT and *Asah1*^fl/fl^/SM^wt^ mice (yellow). In contrast, Cre was only detected in the arterial SMCs of *Asah1*^fl/fl^/SM^Cre^ as shown in co-localization of Cre and CER with SM22-α (Supplementary Fig. S1D). It is clear that *Asah1* gene was deleted in arterial SMCs of *Asah1*^fl/fl^/SM^Cre^ mice.

### Enhanced aortic calcification and SMC phenotypic transition in *Asah1*^fl/fl^/SM^Cre^ mice

**Figure 1:**
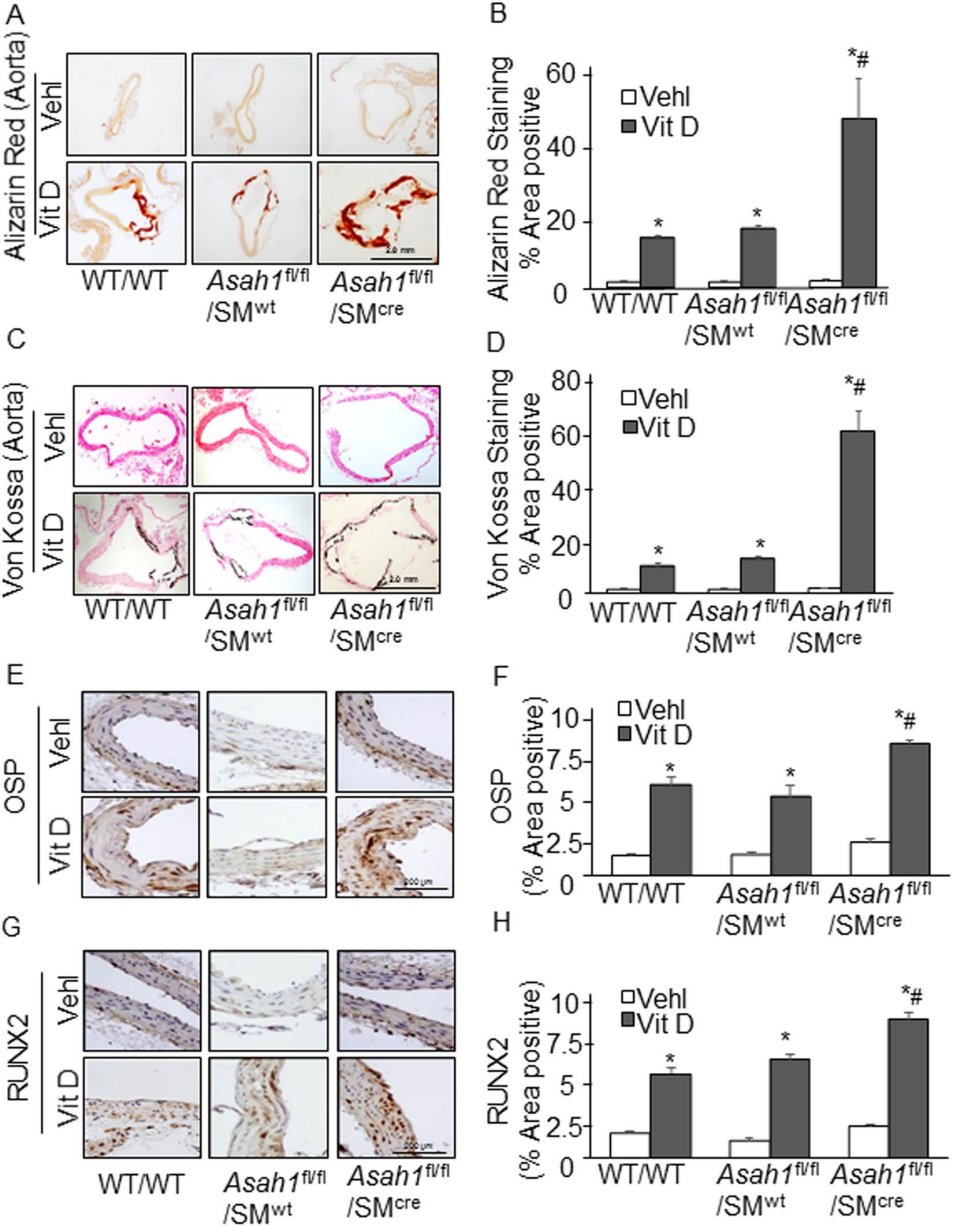
Aortic calcification and smooth muscle phenotype changes in SM-specific *Asah1* KO mice. Representative images of aortic sections stained by (**A**) Alizarin Red S (red color) and (**C**) Von Kossa (black color) staining showed that aorta of *Asah1*^fl/fl^/SM^Cre^ mice exhibited more calcification compared with littermates (*Asah1*^fl/fl^/SM^wt^ and WT/WT). (**B**,**D**) Summarized data in the bar graph clearly revealed that lysosomal acid ceramidase (*Asah1* gene) contribute to the development of AMC. Representative immunohistochemical images from the aorta and quantitative analysis shows that immunostaining of osteogenic markers. (**E,F**) OSP (brown stain) and (**G,H**) RUNX2 (brown stain) significantly increased in the aortic media of Vit D-treated *Asah1*^fl/fl^/SM^Cre^ mice compared to their littermates. Smooth muscle cell (SM); Osteopontin (OSP); Runt-related transcription factor 2 (RUNX2); AMC (arterial medial calcification). Data are shown as means ± SEM, (n = 6). *P < 0.05 vs. WT/WT Vehl; ^#^P < 0.05 vs. WT/WT Vit D group by two-way ANOVA followed by Duncan’s test.

To determine whether *Asah1* gene deletion in SMCs leads to AMC, we employed *Asah1*^fl/fl^/SM^Cre^ mice and their floxed and WT/WT littermates. We induced disordered/imbalanced mineral homeostasis in mice via subcutaneous (s.c) injection of Vit D at a dosage of 500,000 IU/kg/day for 4 consecutive days. It was found that *Asah1* gene deletion in SMCs markedly augmented aortic medial calcification relative to their littermates treated with high doses of Vit D (maximal increase in blood calcium level by ~45%). As shown in Fig. 1A,C, both Alizarin Red S and Von Kossa staining showed that the aorta of *Asah1*^fl/fl^/SM^Cre^ mice exhibited more calcification compared with littermates (*Asah1*^fl/fl^/SM^wt^ and WT/WT). Summarized data in bar graph as shown in Fig. 1B,D clearly revealed that lysosomal Ac deficiency contributes to the development of AMC in the aorta.

In addition, the expression of osteogenic markers viz matrix proteins e.g., osteopontin (OSP) and “bone” transcription factors (e.g., RUNX2) increased considerably in the aortic medial wall of *Asah1*^fl/fl^/SM^Cre^ mice treated with Vit D than their littermates (*Asah1*^fl/fl^/SM^wt^ and WT/WT controls) (Fig. 1E,G). These immunostaining results were summarized in their corresponding individual bar graphs, clearly showing that SM-specific *Asah1* gene deletion significantly enhanced the phenotypic transition to osteogenic status (Fig. 1F,H).

### Coronary AMC and smooth muscle phenotype changes in the coronary arterial wall of *Asah1*^fl/fl^/SM^Cre^ mice

**Figure 2:**
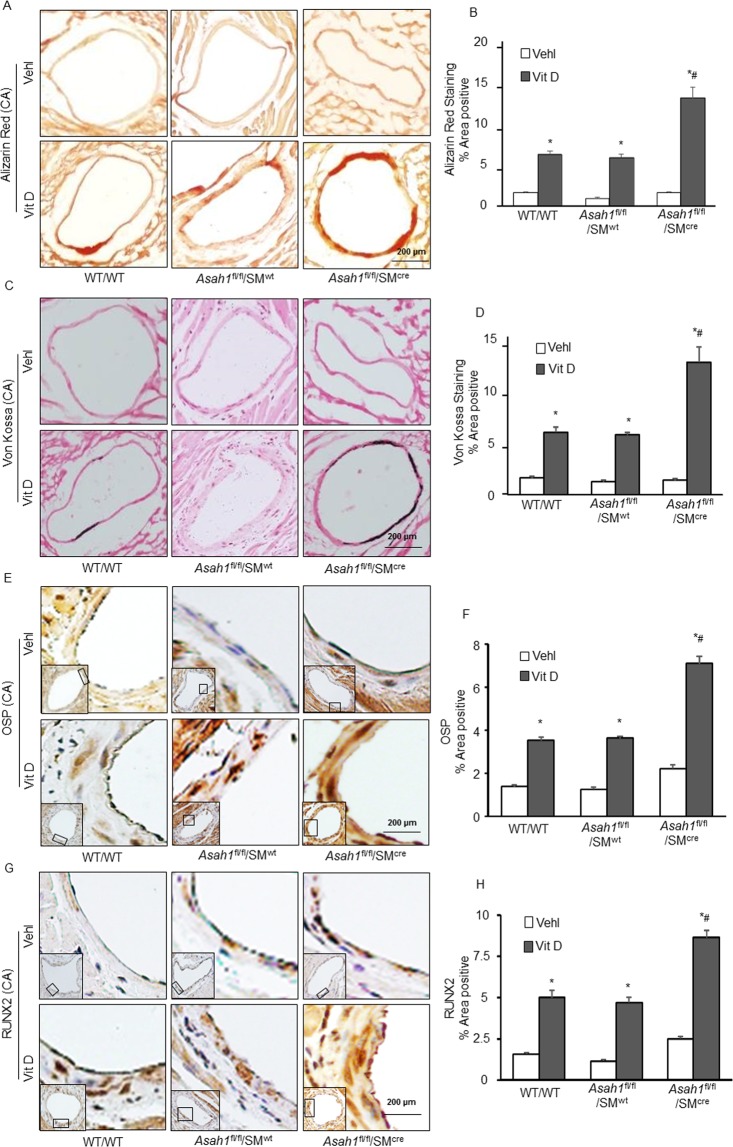
Coronary AMC and smooth muscle phenotype changes in SM-specific *Asah1* KO mice. Representative images of coronary artery sections stained by (**A**) Alizarin Red S (red color) and (**C**) Von Kossa (black color) staining to visualize calcification in the coronary arterial media. (**B,D**) Bar graphs show significant increase in AMC due to *Asah1* gene deletion in *Asah1*^fl/fl^/SM^Cre^ during Vit D treatment as compared to their littermates (*Asah1*^fl/fl^/SM^wt^ and WT/WT), (n = 6). (**E,G**) Representative immunohistochemical images observed that OSP and RUNX2 (brown stain) significantly increased in the coronary arterial wall of Vit D-treated *Asah1*^fl/fl^/SM^Cre^ mice compared to their littermates. (**F,H**) Bar graphs show that SM-specific *Asah1* gene deletion induced phenotypic transition in arterial medial SMCs. Smooth muscle cell (SM); Osteopontin (OSP); Runt-related transcription factor 2 (RUNX2). Data are shown as means ± SEM, (n = 5). *P < 0.05 vs. WT/WT Vehl; ^#^P < 0.05 vs. WT/WT Vit D group by two-way ANOVA followed by Duncan’s test.

We also observed coronary arterial wall calcification pattern in *Asah1*^fl/fl^/SM^Cre^ mice (Fig. 2A,C) to that seen in the aortic medial wall. The summarized data are depicted in the bar graph (Fig. 2B,D), showing significant increase in AMC as detected by Alizarin Red S and Von Kossa staining. Also, immunostaining of the osteogenic markers like OSP (Fig. 2E) and RUNX2 (Fig. 2G) were markedly increased in the coronary arterial wall of *Asah1*^fl/fl^/SM^Cre^ mice treated with Vit-D compared to their littermates (*Asah1*^fl/fl^/SM^wt^ and WT/WT). As shown in the bar graph of Fig. 2F,H, it is clear that phenotypic transition in arterial medial SMCs with *Asah1* gene deletion was associated with increased AMC in *Asah1*^fl/fl^/SM^Cre^ mice, and the OSP, RUNX2 expression was significantly upregulated.

### Reduced lysosome-MVBs interactions and increased sEV markers in the arterial medial wall of Vit D -treated SM-specific *Asah1* KO mice

**Figure 3:**
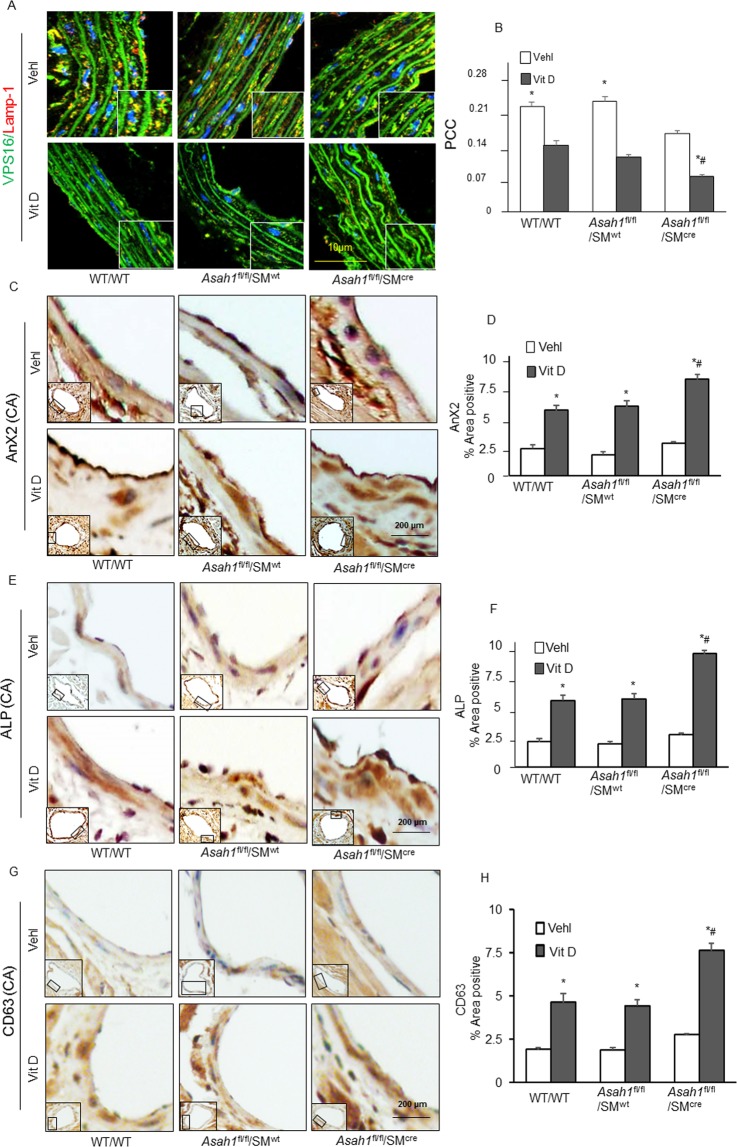
sEV and MVB changes in SM-specific *Asah1* KO. (**A**) Representative confocal microscopic images showed that co-localization of MVBs (VPS16, green) and lysosomes (lysosome marker, Lamp-1, red) in aortic SMCs was much lower in the aortic media of *Asah1*^fl/fl^/SM^Cre^ mice than their littermates (*Asah1*^fl/fl^/SM^wt^ and WT/WT) receiving Vit D injection. (**B**) The co-localization coefficient (PCC) in *Asah1*^fl/fl^/SM^Cre^ mice was significantly reduced as shown in the bar graph. Representative immunohistochemical images showed that sEV and mineralization makers. (n = 6) (**C**) AnX2 (brown stain), (**E**) ALP (brown stain) and (**G**) CD63 (brown stain), (n = 3) staining were significantly increased in the coronary arterial media of Vit D-treated *Asah1*^fl/fl^/SM^Cre^ mice than their littermates. Summarized data showing area percentage of (**D**) AnX2, (**F**) ALP and (**H**) CD63 positive staining in coronary arterial wall, (n = 5–6). Data are shown as means ± SEM. Annexin-II: AnX2, Alkaline phosphatase: ALP, Pearson correlation coefficient: PCC. *P < 0.05 vs. WT/WT Vehl; ^#^P < 0.05 vs. WT/WT Vit D group by two-way ANOVA followed by Duncan’s test.

Given the role of sEVs or microvesicles in the development of tissue calcification, we investigated whether SM-specific *Asah1* gene deletion alters lysosome-MVB fusion which led to release of sEVs in AMC. Sphingolipids such as CER or its metabolites are reported to participate in sEV biogenesis, sorting intraluminal vesicles (ILVs) into MVBs, membrane budding of sEVs into MVBs and their fusion to membrane for release of sEVs^24,35,36^. In the aortic medial wall of *Asah1*^fl/fl^/SM^Cre^ mice treated with Vit D, co-localization of MVBs (VPS16, green) and lysosomes (Lamp-1, red) in SMCs was significantly reduced compared to their littermates (*Asah1*^fl/fl^/SM^wt^, WT/WT) (Fig. 3A). The co-localization coefficient (PCC) of both markers in *Asah1*^fl/fl^/SM^Cre^ mice were significantly decreased as depicted in the bar graph (Fig. 3B). This indicates that more MVBs could not be digested via lysosomes, thus enhancing sEV secretions. Immunohistochemically, we indeed found significant increase in AnX2 (Fig. 3C), ALP (Fig. 3E) and CD63 (3G) staining as sEV and mineralization markers in the coronary arterial wall of Vit D-treated *Asah1*^fl/fl^/SM^Cre^ mice than their littermates (*Asah1*^fl/fl^/SM^wt^ and WT/WT) as shown in the bar graphs (Fig. 3D,3F,3H). The results depict that lysosomal Ac and sphingolipid/CER pathway is important in controlling lysosome fusion with MVBs. SM-specific *Asah1* gene deletion increased CER level in lysosomes which may result in reduced lysosome-MVB interactions, and increased fusion of MVBs with plasma membrane leading to enhanced release of sEVs.

### Increased calcification and phenotypic switch in the *Asah1*^fl/fl^/SM^Cre^ CASMCs *in vitro*

**Figure 4:**
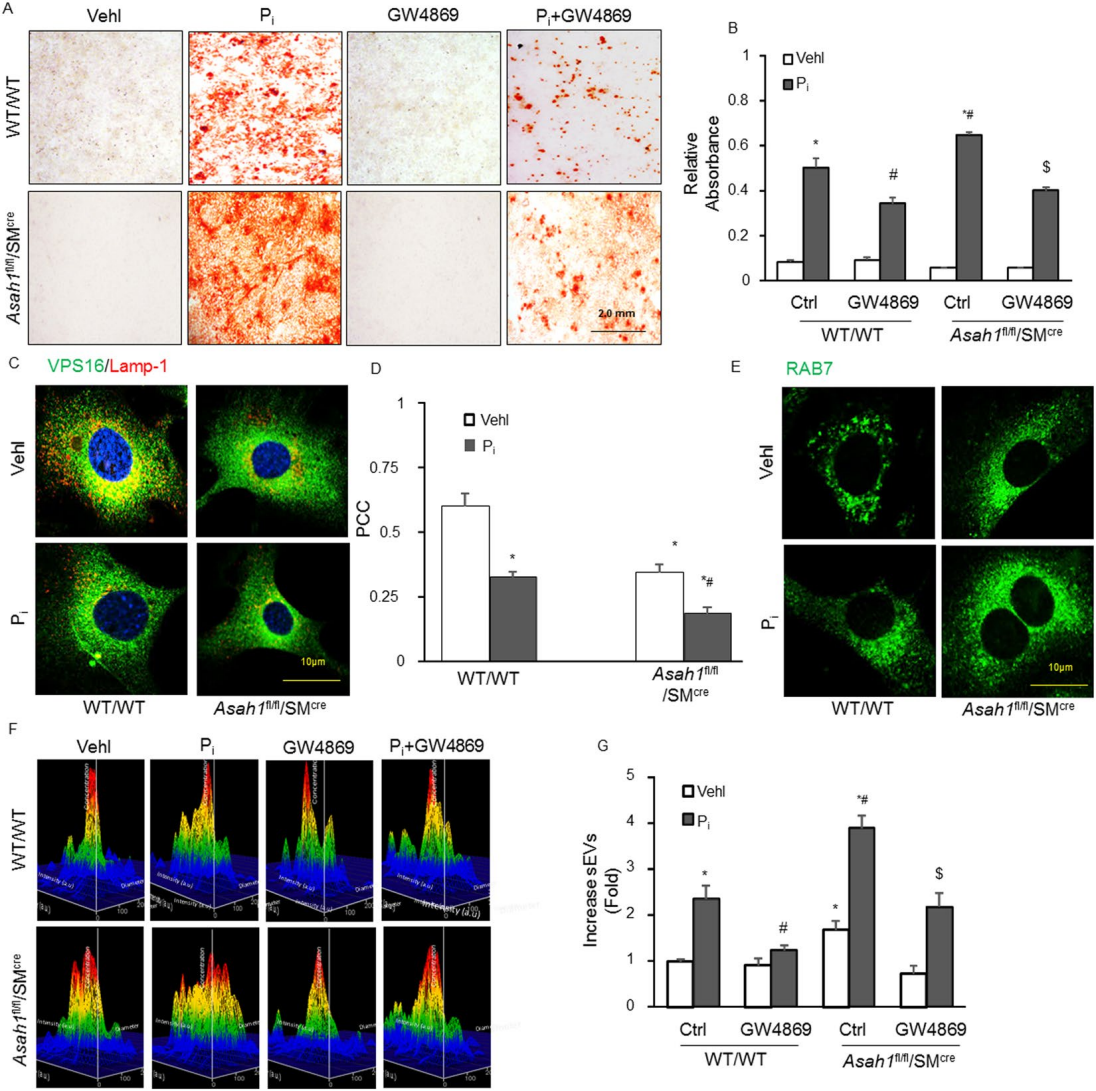
Calcification, lysosome-MVBs interactions and sEV excretion in the SM-specific *Asah1* gene deleted CASMCs *in vitro*. (**A**) Representative images showed calcium deposition in P_i_ and GW4869 treated *Asah1*^fl/fl^/SM^Cre^ CASMCs. (**B**) Summarized bar graph showed GW4869 significantly decreased P_i_-induced calcium deposition, (n = 3). **(C)** Representative confocal images showed co-localization of VPS16 (green) and Lamp-1(red) in CASMCs. (**D**) Bar graph shows significant decrease in co-localization of VPS16/Lamp-1 in P_i_-treated *Asah1*^fl/fl^/SM^Cre^ CASMCs, n = 6. (**E**) Representative photomicrographs showed immunofluorescent images of RAB7 indicating markedly increased RAB7 immunostaining in P_i_ treated *Asah1*^fl/fl^/SM^Cre^ CASMCs, (n = 3). **(F)** Representative images showed sEV release in P_i_ and GW4869 treated *Asah1*^fl/fl^/SM^Cre^ CASMCs. (**G**) Bar graphs shows GW4869 significantly decreased P_i_-induced sEV release in CASMCs of SM-specific *Asah1* KO mice, (n = 3). Data are shown as means ± SEM. Smooth muscle cell (SM); Phosphate: P_i,_ PCC: Pearson correlation coefficient. *P < 0.05 vs. WT/WT Vehl group; ^#^P < 0.05 vs. WT/WT P_i_ group; ^$^P < 0.05 vs*. Asah1*^fl/fl^/SM^Cre^ P_i_ group by two-way ANOVA followed by Duncan’s test and one-way ANOVA followed by Tukey’s test.

To validate our *in vivo* results, we further examined the impact of Cre-mediated *Asah1* gene deletion in CASMCs on vascular calcification. In CASMCs, we determined the impact of the *Asah1* gene deletion in high phosphate (P_i_)-stimulated calcification model. As depicted in Fig. 4A, P_i_ treatment in CASMCs isolated from *Asah1*^fl/fl^/SM^Cre^ mice increased calcium deposition compared to WT/WT cells whileas GW4869, sEV release inhibitor, significantly decreased P_i_-induced calcification in these cells (Fig. 4B) as indicated by the formation of Alizarin Red-stained nodules suggesting that sEV release inhibition may prevent development of AMC in these cells.

**Figure 5:**
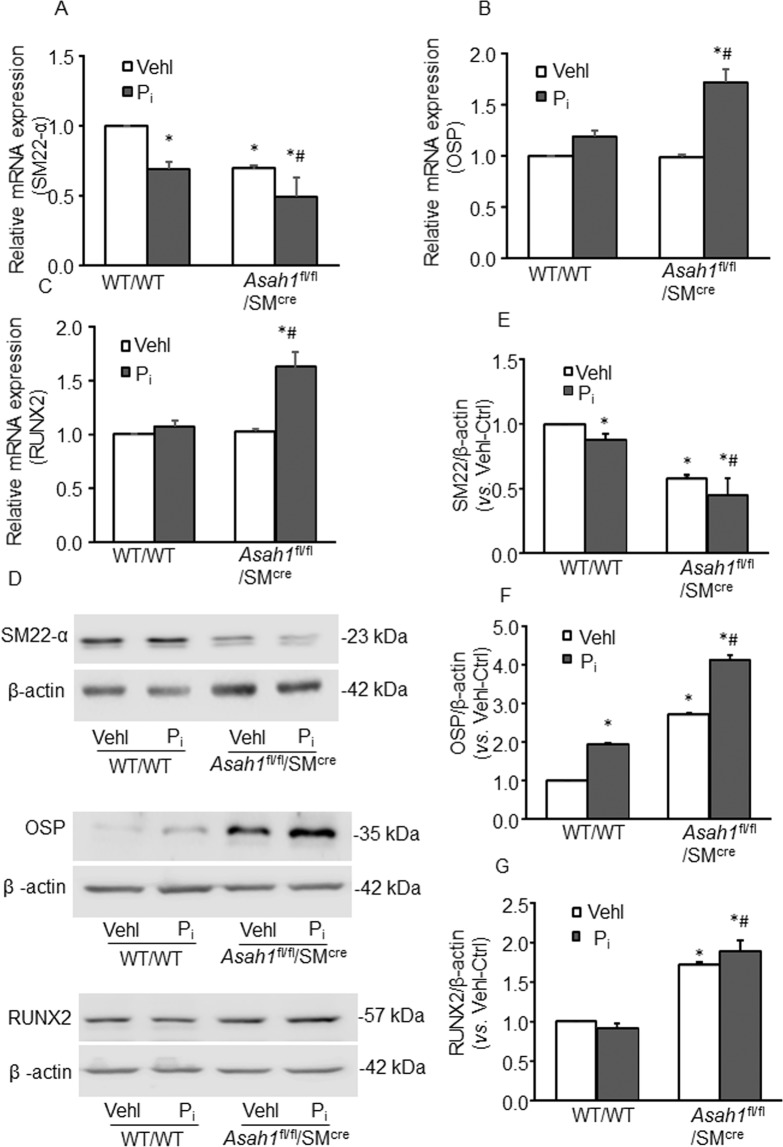
Smooth muscle phenotype changes in SM-specific *Asah1* KO CASMCs *in vitro*. Effects of Ac gene deletion on the mRNA expression of (**A**) SM22-α (**B**) OSP and (**C**) RUNX2 in P_i_-treated CASMCs by RT-PCR, (n = 3). (**D**) Representative western blot analysis showing the effects of Ac gene deletion on SM22-α, OSP and RUNX2 protein expression. Summarized data shows the changes in the protein expression of **E**. SM22-α, (**F**) OSP and (**G**) RUNX2, (n = 4–6). Data are shown as means ± SEM. Smooth muscle cell (SM); Phosphate: P_i_. *P < 0.05 vs. WT/WT Vehl group; ^#^P < 0.05 vs. WT/WT P_i_ group by two-way ANOVA followed by Duncan’s test.

By analyzing SMC phenotype markers, we observed that *Asah1* gene deletion in SMCs induced the phenotypic switch to osteogenic upon P_i_ treatment in CASMCs, as shown by decreased SM22-α (SMC marker) (Fig. 5A), increased OSP (Fig. 5B) and RUNX2 (Fig. 5C) expression by RT-PCR. Also, western blot analysis (Fig. 5D) showed similar trend as shown in the bar graph (Fig. 5E–G). The changes were more prominent with Ac gene deletion in P_i-_treated *Asah1*^fl/fl^/SM^Cre^ CASMCs as compared to WT/WT cells. These results indicated phenotypic transition in CASMCs with *Asah1* gene deletion may be associated with increased AMC, showing augmented expression of OSP and RUNX2.

### Reduced lysosome-MVBs interactions and increased sEVs secretion in the *Asah1*^fl/fl^/SM^Cre^ CASMCs *in vitro*

Immunostaining showed enhanced co-localization of MVB marker (VPS16, green) and lysosome marker (Lamp-1, red) in WT/WT CASMCs, which indicates MVBs fusion with lysosomes. In the absence of high P_i_, CASMCs with *Asah1* gene deletion had largely decreased co-localization of VPS16 vs. Lamp-1 (as shown by fewer yellows dots) in comparison to WT/WT cells, whereas P_i_ exposure decreased co-localization of both markers in CASMCs from WT/WT as well as *Asah1*^fl/fl^/SM^Cre^ cells (Fig. 4C). The bar graphs shows the co-localization coefficient (PPC), with a markedly reduced lysosomal and MVBs interaction in P_i_ -treated *Asah1*^fl/fl^/SM^Cre^ CASMCs in comparison to WT/WT cells (Fig. 4D). Further, we used RAB7 (green, color), a late endosomal marker to observe change in the vesicles, we found the RAB7 immunostaining was markedly increased both in Vehicle and P_i_-treated *Asah1*^fl/fl^/SM^Cre^ CASMCs in comparison to WT/WT cells indicating more MVB formation with *Asah1* gene deletion (Fig. 4E).

Using a nanoparticle tracking analysis system, we found that secretion of sEVs (<200 nm) from CASMCs markedly increased by P_i_, as depicted by representative 3-D histograms in Fig. 4F (increased particles <200 nm in P_i_ treated CASMCs from *Asah1* gene KO mice). A bar graph of vesicle counts of <200 nm (Fig. 4F) showed that sEVs from CASMCs of *Asah1* gene KO mice significantly increased. Also, we treated only *Asah1*^fl/fl^/SM^Cre^ CASMCs with GW4869 which significantly decreased sEVs secretion as compared to both Vehicle as well as P_i_-treated *Asah1*^fl/fl^/SM^Cre^ CASMCs as shown in Fig. 4G. These results suggested that even without P_i_ stimulation sEV release increased from SMCs with *Asah1* gene deletion, which may contribute to arterial medial calcification.

### Characterization of lysosomal Ca^2+^ release via TRPML1 channel activation in CASMCs using GCaMP3-ML1

**Figure 6:**
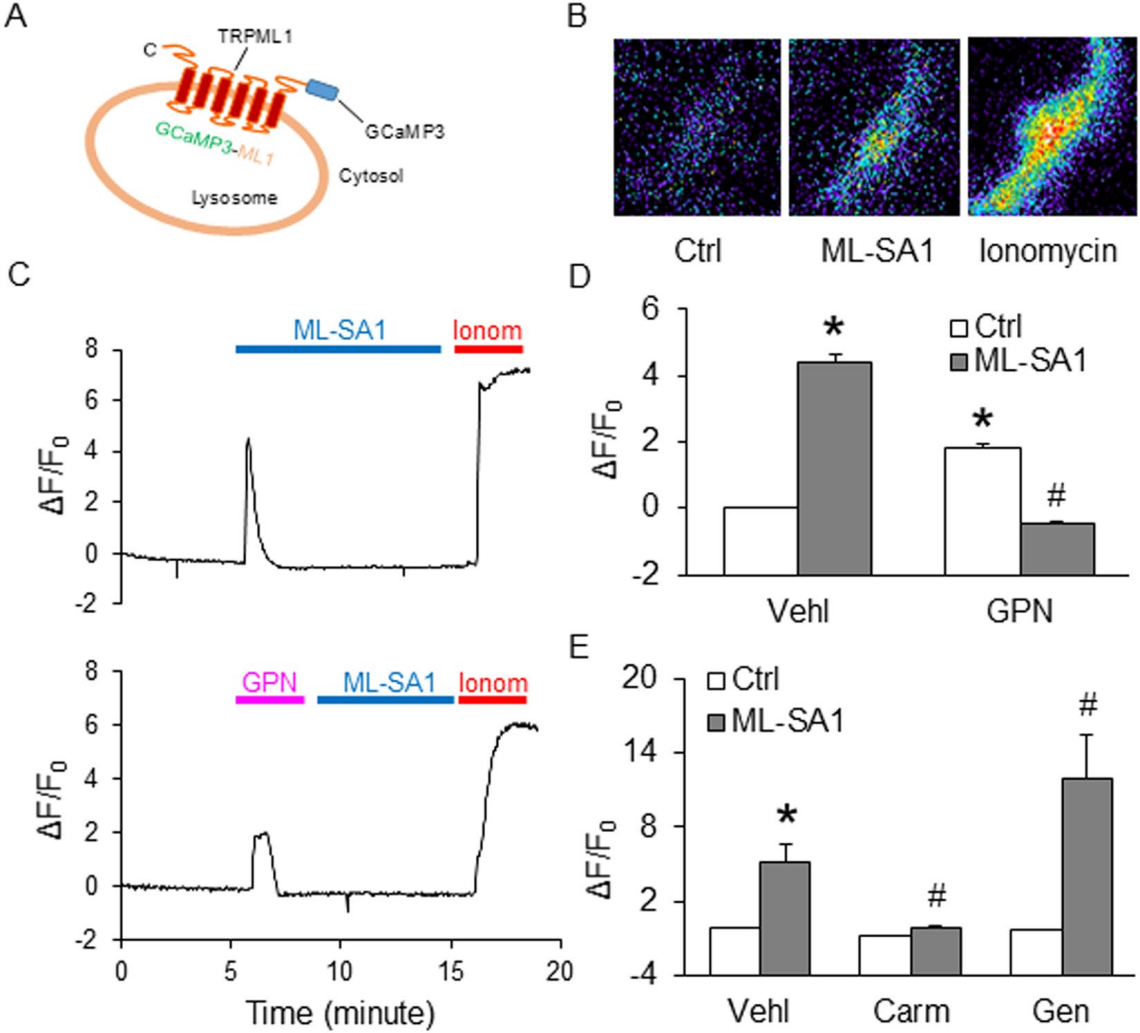
Characterization of lysosomal Ca^2+^ release via TRPML1 channel activation in CASMCs using GCaMP3-ML1. (**A**) The strategy of GCaMP3-TRPML1 (GCaMP3-ML1) fusion. GCaMP3 is fused to the N-terminus of TRPML1. (**B**) Representative micrographs showing the changes of GCaMP3 fluorescence upon bath application of ML-SA1 and ionomycin to GCaMP3-ML1-transfected CASMCs. (**C,D**) Rapid increases in GCaMP3 fluorescence was induced by ML-SA1 (measured as the change of GCaMP3 fluorescence ∆F over basal fluorescence F0; ∆F/F0) in CASMCs transfected with GCaMP3-ML1. Subsequent application of GPN (200 µM) induced smaller responses than ML-SA1. On the other hand, ML-SA1 induced small responses in GCaMP3-ML1-expressing CASMCs that had received an application of GPN. The application of ionomycin (1 µM) induced maximal responses. (**E**) ML-SA1 had no effects on GCaMP3 fluorescence in GCaMP3-ML1-expressing CASMCs after pre-treatment with carmofur (Carm, 1 µM) for 30 minutes, however geniestein (Gen, 20 µM) pretreatment enhanced ML-SA1-induced Ca^2+^ release through TRPML1 channel, (n = 4). Data are shown as means ± SEM. GPN: glycyl-L-phenylalanine 2-naphthylamide; Ionom: ionomycin. *p < 0.05 vs. Ctrl-Vehl group. ^#^p < 0.05 vs. pre-treatment with ML-SA1 group by two-way ANOVA followed by Duncan’s test.

GCaMP3, a genetically encoded calcium ion indicator attached to the cytoplasmic amino-terminal domain of TRPML1 with single-wavelength was used to determine TRPML1-mediated lysosomal Ca^2+^ release in intact CASMCs (Fig. 6A). Further, we observed the effect of TRPML1 channel agonist ML-SA1, carmofur (a selective Ac inhibitor) or glycyl-L-phenylalanine 2-naphthylamide (GPN) on transfected CASMCs to analyze the specificity of the GCaMP3-ML1 fluorescence signal. GPN, also a substrate for cathepsin C which induces the osmotic lysis of lysosomes was used to determine the reduction of GCaMP3 detected Ca^2+^ signals by emptying lysosome Ca^2+^ storage. GCaMP3 fluorescence signal (F470) was constantly monitored in CASMCs by using fluorescent microscopic imaging system as shown in Fig. 6B. A marked increase of GCaMP3 fluorescence was observed after the addition of ML-SA1 in CASMCs while as addition of GPN induced less GCaMP3 fluorescence signal in these CASMCs (Fig. 6C). However, GCaMP3 fluorescence was markedly increased in CASMCs after the early addition of GPN followed by no change of GCaMP3 fluorescent signal induced by late addition of ML-SA1. The quantification data of fluorescence was summarized in Fig. 6D. We also observed a decrease in Ca^2+^ release by carmofur, a selective Ac inhibitor, while a significant increase by genistein (Gen) through ML-SA1 induced TRPML1 channel as compared to the vehicle (Fig. 6E). With these results, it is confirmed that GCaMP3 detected Ca^2+^ release on the lysosomal membrane of CASMCs was through TRPML1 channels present on lysosome membrane. These data also confirmed that activation of Ac contributed to the TRPML1 channel mediated Ca^2+^ release.

### Effect of TRPML1 activator on lysosome-MVBs interactions and sEVs secretion in the CASMCs *in vitro*

**Figure 7:**
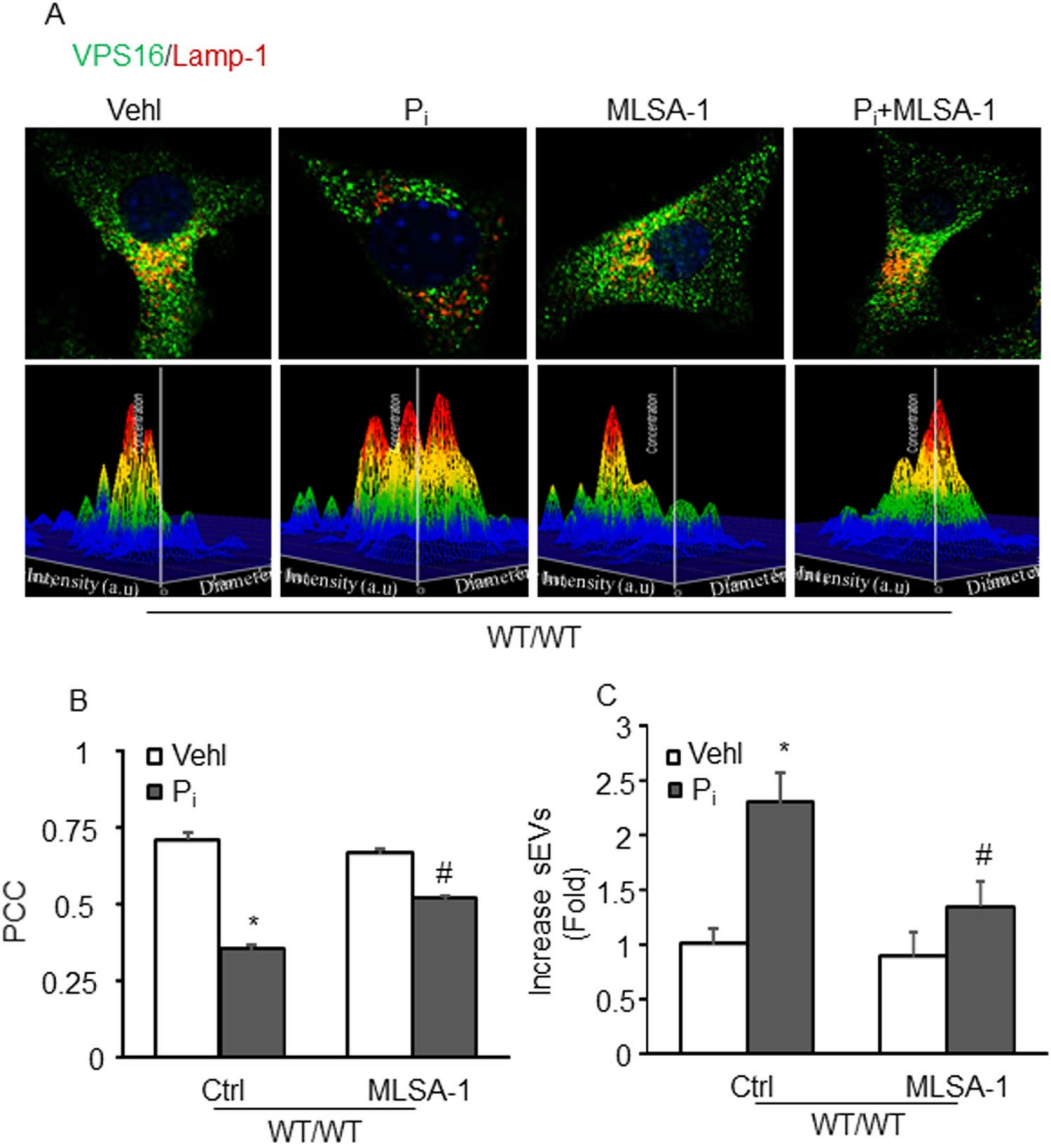
Effect of TRPML1 channel activator on lysosome-MVBs interactions and sEV secretion in the CASMCs *in vitro*. (**A**) Representative confocal images showed co-localization of VPS16 (green) and Lamp-1(red), and sEV release in wild type CASMCs (**B,C**) Bar graph shows ML-SA1 significantly increased VPS16-Lamp-1 co-localization and decreased sEV release in P_i_-treated WT/WT CASMCs, n = 3. Data are shown as means ± SEM. Phosphate: P_i,_ PCC: Pearson correlation coefficient. *P < 0.05 vs. WT/WT Vehl group; ^#^P < 0.05 vs. WT/WT P_i_ group by two-way ANOVA followed by Duncan’s test and one-way ANOVA followed by Tukey’s test.

Immunostaining showed augmented co-localization of MVB marker (VPS16, green) and lysosome marker (Lamp-1, red) in WT/WT CASMCs, which indicates MVBs fusion with lysosomes. With high P_i_, these WT/WT CASMCs showed significantly decreased co-localization of VPS16 vs. Lamp-1 (as shown by fewer yellows dots) in comparison to vehicle WT/WT cells (Fig. 7A). However, the co-localization of VPS16 vs. Lamp-1 in P_i_ treated WT/WT CASMCs was markedly increased by ML-SA1. The bar graph shows the co-localization coefficient (PPC) (Fig. 7B). Also, nanoparticle tracking analysis of these wild type CASMCs showed secretion of sEVs were significantly increased by P_i_, whileas ML-SA1 significantly decreased P_i_-induced sEVs secretion in these cells as depicted by representative 3-D histograms (Fig. 7A) and bar graph in Fig. 7C.These data suggest that TRPML1 Ca^2+^ signaling may be associated with lysosome-MVB interactions and sEV secretion in CASMCs.

### Role of Ac in lysosomal Ca^2+^ release via TRPML1 channel activation

**Figure 8:**
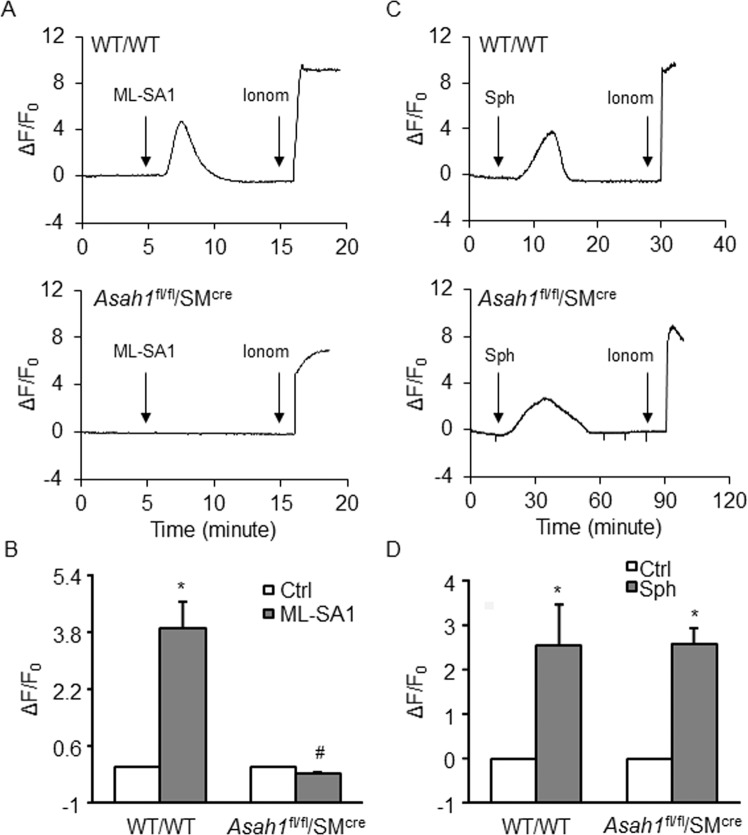
Effect of Ac gene deletion on lysosomal Ca^2+^ release via TRPML1 channel activation in CASMCs. (**A**) ML-SA1 (20 µM) induced remarkable increases in GCaMP3 fluorescence in WT/WT CASMCs transfected with GCaMP3-ML1, which was decreased with Ac gene deletion in *Asah1*^fl/fl^SM^Cre^ CASMCs. (**B**) Summarized data shown in bar graph, (n = 5) (**C**) Sph (20 µM) induced remarkable increases in GCaMP3 fluorescence in WT/WT CASMCs transfected with GCaMP3-ML1. Also, Sph markedly increased GCaMP3 fluorescence in GCaMP3-ML1-expressing *Asah1*^fl/fl^SM^Cre^ CASMCs. The application of ionomycin (1 µM) induced maximal responses. (**D**) Summarized data showing responses in CASMCs transfected with GCaMP3-ML1 induced by Sph, (n = 4). Data are shown as means ± SEM. Ctrl: control; Sph: sphingosine; Ionom: ionomycin. *p < 0.05 vs. WT/WT Ctrl group. ^#^p < 0.05 vs. WT/WT ML-SA1 group by two-way ANOVA followed by Duncan’s test and one-way ANOVA followed by Tukey’s test.

It has been reported that sphingolipids regulates TRPML1 channel activity, thus we tested whether Ac gene deletion cause alteration in the opening of TRPML1 channels in CASMCs. We measured the ceramide and sphingosine levels by HPLC-MS/MS analysis and observed that total ceramide was increased by ~3.2 folds with Ac gene deletion in *Asah1*^fl/fl^ SM^Cre^ CASMCs as compared to wild type cells, whereas there was nonsignificant increase in sphingosine by ~0.32 folds. Increased levels of different ceramides was observed C14 (~0.6 folds) C16 (~3.2 folds), C18 (~9.7 folds), C20 (~3.9 folds), C22 (~4.9 folds) and C24 (~4.4 folds) in *Asah1*^fl/fl^ SM^Cre^ CASMCs as compared to wild type cells. (Data not shown). As shown in Fig. 8A, Ac gene deletion remarkably decreased ML-SA1 induced Ca^2+^ release through TRPML1 channels as compared to WT/WT CASMCs, which was further quantified by GCaMP3 fluorescent signal (Fig. 8B). We also analyzed the effect of sphingosine (Sph), a product of Ac-dependent CER metabolism on TRPML1 channel activity and observed that Sph markedly induced TRPML1 channel activity. While there was no effect of Ac gene deletion on Sph-induced Ca^2+^ release in CASMCs, which revealed that Sph-induced Ca^2+^ release was specific to TRPML1. Thus Ac metabolite, Sph generated from CER may be activator of TRPML1 channels (Fig. 8C). Ca^2+^ release through TRPML1 channel was also quantified by GCaMP3 fluorescent signal (Fig. 8D), confirming that smooth muscle specific Ac gene deletion blocked ML-SA1-induced Ca^2+^ release from lysosomes, but did not have effects on Sph-induced Ca^2+^ release from lysosomes. Moreover, by determining the co-localization of lysosome marker (Lamp-1, Red) vs. Sphingosine (Sph, green) we found that sphingosine staining in *Asah1*^fl/fl^ SM^Cre^ CASMCs was much lower as compared to WT/WT type cells, indicating that *Asah1* gene deletion indeed decreased lysosomal Sph levels (Supplementary Fig. S2A,B).

### Effects of Ac-associated sphingolipids on TRPML1 channel currents

**Figure 9:**
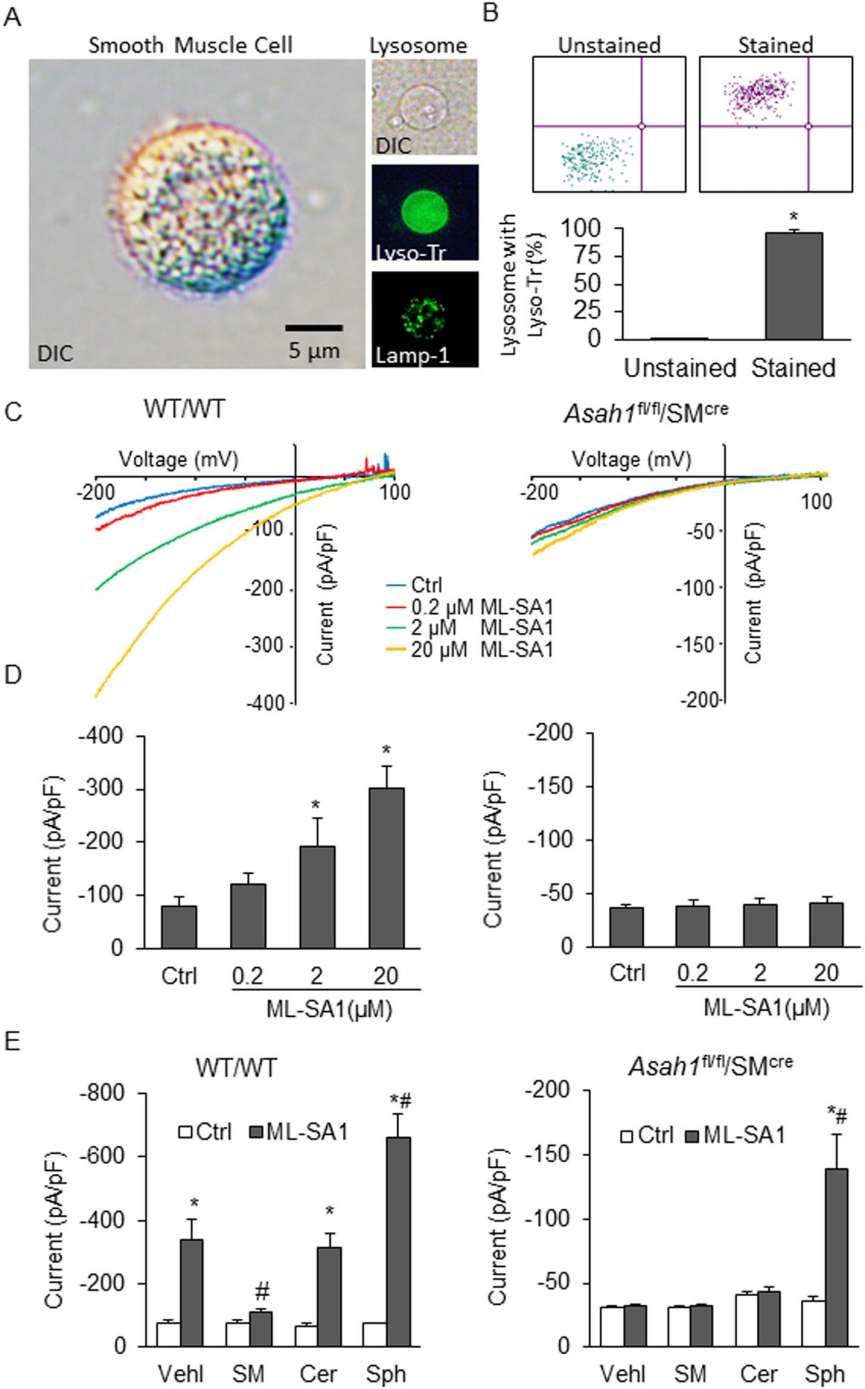
Effects of different sphingolipids on TRPML1 channel currents. (**A**) Representative images of (1) DIC picture of suspended CASMCs, (2) DIC picture of the isolated lysosome, and (3) isolated lysosome stained with LysoTracker. (**B**) The purity of isolated lysosomes detected by flow cytometry. *p < 0.05 vs. Unstained group or Ctrl group. (**C**) Representative whole-lysosome currents enhanced by ML-SA1 in WT/WT CASMCs, but it had no effect on *Asah1*^fl/fl^SM^Cre^ CASMCs. (**D**) ML-SA1 enhanced TRPML1 channel activity in a concentration-dependent manner in WT/WT CASMCs. (**E**) Summarized data of the effects of various sphingolipids on whole-lysosome currents in both WT/WT and *Asah1*^fl/fl^SM^Cre^ CASMCs. Data are shown as means ± SEM. Ctrl: control; Vehl: vehicle; SM: sphingomyelin; Cer: ceramide; Sph: sphingosine. *p < 0.05 vs. Ctrl-Vehl group. ^#^p < 0.05 vs. pre-treatment with ML-SA1 group by two-way ANOVA followed by Duncan’s test and one-way ANOVA followed by Tukey’s test.

Further, we determined the role of Ac associated sphingolipids (sphingomyelin, CER, and Sph) on TRPML1 channel-mediated lysosomal Ca^2+^ release by using whole-lysosome patch clamp recording. Using differential interference contrast (DIC) microscopy, lysosomes isolated from CASMCs were confirmed and compared to CASMCs in size (Fig. 9A). Isolation of lysosomes was validated using LysoTracker which was confirmed by green fluorescence in LysoTracker loaded lysosomal lumen indicating its low pH. Besides, flow cytometry confirmed the high purity of isolated lysosomes, which was revealed by the quantification of flow cytometry data (Fig. 9B).

To characterize the TRPML1 channel currents properties in isolated lysosomes, we used the whole-lysosome patch clamp approach and observed the Ca^2+^ currents induced by bath application of ML-SA1

in a dose-dependent manner in lysosomes of WT/WT CASMCs, whereas Ac gene deletion caused inhibition of ML-SA1-induced Ca^2+^ channel activation in the *Asah1*^fl/fl^/SM^Cre^ CASMCs (Fig. 9C,9D). In bath solution of 20 µM ML-SA1, the whole-lysosome current was −300.45 ± 42.57 pA observed with positive reversal potential at −200 mV. These data confirmed the TRPML1 channel function in lysosomes isolated from CASMCs. In addition, we analyzed the effect of sphingolipids associated with Ac on TRPML1 channel currents of these isolated lysosomes. We found that sphingomyelin blocked Ca^2+^ currents induced by ML-SA1 in WT/WT CASMCs (Fig. 9E). Also, we did not observe any effect of CER, an Ac substrate, on ML-SA1 induced TRPML1 channel-mediated Ca^2+^ currents, however, Sph (Ac product) enhanced activation of TRPML1 channel by ML-SA1. Therefore, these results suggested that Sph production may be decreased due to Ac inhibition thereby reducing TRPML1 channel activity. It is likely that lysosomal CER accumulation induced by decreased Ac activity may increase sphingomyelin which inturn suppressed TRPML1 channel activity.

## Discussion

The present study revealed for the first time that lysosomal Ac plays a key role in sEV release, osteogenic phenotype transition and resulting in mineral deposition in arterial SMCs that contribute to AMC. Our results also show that lysosomal Ac and associated CER signaling pathway may control the fate of MVBs via its regulatory action on TRPML1 channel activity and subsequent lyososome-MVB interaction, which leads to enhanced sEV release from SMCs that influence the AMC development.

We first revealed that Ac gene deletion in SMCs using *Asah1*^fl/fl^/SM^Cre^ mice greatly increased the calcification in aortic and coronary arterial medial wall in comparison to their littermates during hypercalcemia caused by high doses of Vit D. In addition, “bone” transcription factors (e.g., RUNX2) and matrix proteins (e.g. osteopontin) have been noticeably increased in the aortic and coronary arterial medial wall of *Asah1*^fl/fl^/SM^Cre^ mice treated with Vit D compared to their littermates (*Asah1*^fl/fl^/SM^wt^ and WT/WT). These results indicate that phenotypic transition in arterial SMCs with *Asah1* gene deletion may contribute to the development of AMC. We further investigated whether SM-specific *Asah1* gene deletion alters lysosome-MVB interaction leading to sEV release in AMC. Much lower co-localization of MVBs (VPS16, green) and lysosomes (Lamp-1, red) within aortic medial SMCs was observed in *Asah1*^fl/fl^/SM^Cre^ mice in comparison to their littermates (*Asah1*^fl/fl^/SM^wt^, WT/WT), after treatment with Vit D. Also, in the coronary arterial media expression of CD63, AnX2 (sEV markers) and ALP expression (mineralization marker) were markedly increased upon Vit D-treatment in *Asah1*^fl/fl^/SM^Cre^ mice corresponding than their littermates (*Asah1*^fl/fl^/SM^wt^ and WT/WT). These findings from animal experiments were also validated by cell studies *in vitro* in CASMCs, where we have found that Cre-mediated *Asah1* gene deletion augmented calcium deposition, RAB7, a late endosomal marker and sEV secretion in association with decreased lysosome-MVB interaction as shown by co-localization of VPS16 and Lamp-1 if these cells were exposed to high P_i_ in the culture media. Furthermore, using GW4869, sEV release inhibitor, we observed reduced calcification and sEV secretion. These results indicate that SM-specific *Asah1* gene deletion leads to failure of lysosome-MVB interactions, increasing fusion of MVBs with plasma membrane thereby enhancing release of sEVs from SMCs. This Ac mediated regulation of SMC lysosome function and sEV secretion may represent a novel mechanism in preventing AMC from different pathological stimuli.

With respect to the pathogenesis of AMC, recent studies have reported that calcification development in the arterial media is actively regulated by controlling the osteogenic differentiation of SMCs and matrix mineralization^37–39^. However, the precise mechanisms underlying these processes in AMC are still unknown. A previous study has revealed that GW4869, a neutral sphingomyelinase (N-SMase) inhibitor, completely inhibits apolipoprotein C-I-induced vascular SMC apoptosis and CER generation^40^. GW4869, an N-SMase inhibitor was found to inhibit Ox-LDL-induced apoptosis in SMCs, which was prevented by C2-ceramide. In cultured SMCs, GW4869 markedly decreased calcification induced by Ox-LDL and prevented Msx2 expression up-regulation in these cells, suggesting involvement of N-SMase in Ox-LDL-induced vascular mineralization^41^. Also, GW4869 significantly decreased the exosome release in VSMCs^18^. In pulmonary artery endothelial cells (PAEC), lipopolysaccharide (LPS) enhanced exosome release, and co-culture of these LPS-stimulated PAEC with pulmonary artery smooth muscle cells (PASMC) leads to over proliferation and apoptosis resistance in PASMC. This effect was partially blocked by GW4869^42^. In Wistar rats, GW4896 significantly decreased circulating extracellular vesicles/exosomes 24 hrs post-myocardial infarction, and also recovered left ventricular ejection fraction (EF%) to a greater extent^43^. Another study demonstrated that pre-treatment with GW4869 in RAW264.7 macrophages and wild-type mice significantly decreased release of exosomes and pro-inflammatory cytokines (TNF-α, IL-1β, IL-6) and indicated that blocking exosome generation in sepsis reduces the sepsis-triggered inflammatory response which inturn improves cardiac function and survival^44^.

In patients with calcified plaques there was a direct correlation of increased activity of N-SMase with elevated levels of CER and apoptosis in plaque. In isolated SMCs, it was also found that Ox-LDL-induced apoptosis involves the activation of N-SMase and CER generation^41,45^. In our previous studies, lysosome dysfunction was found to induce the development of calcification of atheromatous plaques and even in arterial medial layer. Therefore, it is reasonable to speculate the role of lysosomal sphingolipid/CER pathway in SMCs as an underlying mechanism for the development of AMC. The present study used genetic intervention to demonstrate that SM-specific deletion of lysosomal Ac (*Asah1* gene) increased calcification in the mouse arterial medial wall. SM-specific deletion of *Asah1* gene blocked the conversion of CER to Sph in SMCs of *Asah1*^fl/fl^/SM^Cre^ mice, resulting in accumulation of CER from sphingomyelin^46^. It has been suggested that lysosomal CER increase may determine the phenotypic changes in SMCs leading to AMC. In this regard, a recent study using human femoral arterial SMCs showed that CER may mediate Ox-LDL-induced matrix mineralization^41^, as a result of augmented N-SMase activity and CER concentrations. Kapustin *et al*. also reported that neutral sphingomyelinase-2 inhibition reduces mineralization in response to osteogenic medium in human CASMCs^18^. However, neither of the above mentioned studies in human VSMC^18,41^ investigated the role of endogenous CER in lysosomes during matrix mineralization. So far there is no direct evidence that shows the contribution of lysosomal CER metabolism to AMC is due to defect of Ac gene. For instance, Song *et al*. demonstrated that TLR4 modulated Ox-LDL-induced vascular calcification through activation of factor kappa B (NK-κB) p65, which was attenuated by NK-κB inhibitor, pyrrolidine dithiocarbamate (PDTC). The inhibition by PDTC was rescued by C2-ceramide treatment, suggesting that TLR4/NF-κB/Ceramide signaling mediates Ox-LDL induced calcification of human VSMCs^47^. Ceramide significantly enhances phenyl epinephrine (PE)-induced vasoconstriction in rat aortas via ER stress/COX-2/PGE2 pathway^48^. Morris *et al*. determined the role of ezrin-radixin-moesin (ERM) proteins as important downstream effectors of sphingosine-1-phosphate (S1P)-induced VSMC matrix mineralization *in vitro*. The study used pharmacological inhibitors such as desipramine for the inhibition of acid sphingomyelinase (ASM) and ceramidase to elucidate the role of sphingolipids *in vitro*, but lacking genetic manipulations and *in vivo* animal model approach in this context^49^. Of course, involvement of various other pathways cannot be excluded, including inflammatory pathways and oxidative stress pathways involving in ceramide-induced vascular complications. In patients with cystic echinococcosis (CE), however, a correlation between *Asah1* gene expression and cystic calcification was observed. The relative expression of *Asah1* gene was low in the calcification group patients with CE^50^. Also, in patients with acid ceramidase deficiency, hematologic manifestations included calcification of the axillary lymph nodes were observed^51^. Our results provided evidence that phenotype change in arterial medial SMCs to osteogenic may contribute to the pathogenesis of AMC. Together these findings provide more evidence in this direction that defect of *Asah1* gene in SMCs may be attributed to the development of AMC.

It is well known that the plasticity is an important feature of SMCs, which allows them to adapt to various environmental stimuli including pressure and injury^52^. Upon various pathological stimuli or injurious challenges, VSMCs undergo phenotypic switch from the contractile phenotype to synthetic or osteogenic^8,53^. In AMC, osteogenic phenotype was found in vascular medial SMCs in both human and laboratory animals, and these medial SMCs mainly contributes to vascular calcification^39,54,55^. Our results revealed that *Asah1* gene deficiency had remarkable effects on Vit D-induced OSP and RUNX2 expression, also SMC specific deletion of *Asah1* gene triggered the phenotypic transition to osteogenic in P_i_ -treated CASMCs, as demonstrated by decreased SM22-α (SMC marker), increased OSP and RUNX2 expression suggesting that lysosomal *Asah1* expression and associated sphingolipid-CER pathway plays an important role in phenotypic transition in SMCs undergoing AMC.

Next, we addressed whether sEV secretion as triggering mechanism of calcification process is modulated by lysosomal Ac-mediated signaling mechanisms in VSMCs. Exact origin of vesicles derived from SMCs remain poorly understood, previously because of their functional similarity to membrane vesicles which are involved in bone mineralization, they were known as multivesicles^37,56^. However, presently SMC derived matrix vesicles are termed as exosomes because of their formation by inverted budding into MVBs and fusion of MVBs with the plasma membrane which was found by fetuin-A uptake and trafficking^57,58^. VSMC-derived vesicles have augmented exosomal markers such as CD63 and AnX2, and their generation and fetuin-A recycling are controlled by SMPD3, a known controller of exosome biogenesis^24^. CD63 protein belongs to transmembrane 4 superfamily (tetraspanin) which is comprised of a short cytoplasmic tail, four transmembrane α-helices and two extracellular loops. CD63 is enriched in late endosomes, cell organelle like lysosomes, secretory vesicles, and in the plasma membrane. At the plasma membrane CD63 interact with cell adhesion molecules like integrins which regulate various cellular signaling pathways including cell adhesion, motility, and survival^59^. Vascular endothelial cells contain secretory organelles, Weibel-Palade bodies (WPB), which has CD63 one of its components. CD63 is known as a marker of the intralumenal vesicles within multivesicular endosomes. It has also been demonstrated that intimal and medial both types of vascular calcifications are associated with significant increase in EVs in the vascular interstitial space, particularly, the sEVs/exosomes (size of 40–100 or to 140 nm in size). Such sEVs are primarily produced and excreted from arterial SMCs^18–20^. There is also increasing evidence that enhanced sEV secretion may trigger SMC phenotype changes^60,61^, the apatite nucleation^62^, and calcifying nidus formation^63^, ultimately leading to extracellular matrix (ECM) mineralization^18,64^. The present study demonstrated that SM-specific *Asah1* gene deletion substantially reduced interactions of lysosome and MVB in aortic medial SMCs as shown by decreased co-localization of VPS16 vs. Lamp-1. This decreased lysosome-MVB interaction may reduce degradation of MVBs by lysosomes and enhanced MVBs fusion with the plasma membrane to release sEVs. Augmented sEVs in arterial interstitial spaces may trigger nidus formation for mineralization^18,20^. Indeed, we found that CD63, AnX2 (sEV markers) and ALP expression (mineralization marker) was augmented in the arterial media of SM-specific *Asah1* gene KO mice. Nanoparticle tracking analysis also revealed that the secretion of sEVs were increased in CASMCs of SM-specific *Asah1* gene KO mice. All these results from *in vivo* and *in vitro* experiments support the view that lysosomal Ac-mediated CER metabolism exerts a gating action on the sEV secretion, which may consequently induce AMC^20,24^. Recently our group revealed that d-ribose induced IL-1β secretion via EVs in podocytes. Also, it was observed that ceramide in podocytes was found to be elevated upon d-ribose stimulation. However, prior treatments of podocyte with acid ceramidase inducer (genistein) or *Asah1* CRISPR/cas9 activation plasmids were found to decrease d-ribose-induced ceramide accumulation, EVs release and IL-1β secretion due to reduced interactions of MVBs with lysosome. These results indicated that d-ribose stimulation lead to the release of inflammasome-derived products such as IL-1β via EVs, in which lysosomal sphingolipid-mediated regulation of lysosome function plays an important role^65^. To our knowledge, the present study provide the first experimental evidence demonstrating the critical role of lysosomal Ac in the regulation of sEV secretion from SMCs and in the development of AMC. Which to our knowledge we have for the first time explored the molecular mechanisms of vascular calcification mediated by the enhanced release of exosomes from arterial smooth muscle cells.

In addition, we also demonstrated that TRPML1 channel activation in SMCs was attributed to the Ac-mediated regulation and subsequent production of Sph. Literature cites that lysosome trafficking is a main regulatory mechanism of autophagic flux, which may depend upon the Ca^2+^ bursts from lysosomes, activating global Ca^2+^ release from the sarcoplasmic reticulum (SR), that may be enough to drive the lysosome movement along microtubules to meet with other cellular vesicles such as endosomes, MVBs, autophagosomes and SR^66–69^. In the present study, we also observed that ML-SA1-induced TRPML1 channel activation increased lysosome-MVB interaction and consequently decreased sEV excretion. However, effect of TRPML1 channel activation on lysosome-MVB interaction and sEV secretion was observed only under P_i_ stimulation. Interestingly, no significant change was observed at baseline line which reveals that TRPML1 channel activation shows its effect under stress conditions. Recently, sphingolipid regulation of TRPML1 channel on lysosomes has been shown to be a crucial mechanism responsible for lysosome trafficking. In this regard, Shen *et al*. reported that sphingomyelins accumulation particularly in Niemann-Pick cell lysosomes inhibited TRPML1 activity and reduced lysosomal Ca^2+^ release, leading to failure of lysosome trafficking^32,33^. Another study in human fibroblasts derived from a control and patient suffering from mucolipidosis type IV (MLIV) demonstrated that both control and MLIV patient human fibroblasts showed immediate calcium response upon release of Sph which was even higher in MLIV patient human fibroblasts. These findings suggest the specificity towards two pore channel 1 (TPC1), and Sph-induced calcium signaling is independent of a loss of TRPML1 function. In fact, in order to maintain acidic compartment calcium concentrations, the loss of TRPML1 might even increase the importance of calcium efflux through TPCs^70^. It is known that Ac deficiency may increase substrate lysosomal CER and even upstream sphingomyelin, but reduces lysosomal Sph or its metabolite, S1P. Some of them were recently shown to alter lysosomal TRPML1 channel activity^71^ and Ca^2+^ release regulating lysosome trafficking^33,72,73^. The present study tested the hypothesis that the role of Ac may be associated with lysosomal TRPML1-mediated Ca^2+^ release. We indeed confirmed that ML-SA1-induced Ca^2+^ release through TRPML1 channels was reduced by *Asah1* gene deletion and that this TRPML1 channel in lysosome failed to activate in CASMCs from *Asah1* gene knockout mice. Interestingly, we showed that reduced lysosomal Ca^2+^ release and suppressed TRPML1 channel activation was reversed by Ac metabolite, Sph, indicating the crucial role of Ac metabolite from CER.

The present study did not try to explore the mechanistic pathway by which sphingolipids regulate TRPML1 channel activity. However, association of sphingolipids with TRPML1 channel activity was previously reported by the some studies. It has been reported that TRPML1 channel activity was modulated by the highly acidic environment in the lysosome^74^. Ca^2+^ release through TRPML1 channel activation was favored by acidic pH of lysosomes, whereas high pH of the extracellular milieu on the plasma membrane inhibits Ca^2+^ influx^75^. Similarly, defective lysosomal acidification results in an imbalance between ASM cleavage of sphingomyelin to CER and Ac consumption of CER, which may lead to increased CER levels and consequent cystic fibrosis^76^. These previous findings have indicated that TRPML1 channel inhibition due to defective lysosomal acidification may be explained by alteration of sphingolipid metabolism. In addition, phosphoinositides (PIPs) as an important regulator of TRPML1 channel activity are also essential for fusion-fission events in intracellular trafficking^74^. Recently, our group demonstrated the role of lysosomal TRPML1 channel activity and exosome release by acid ceramidase in a cell line (podocytes) that mainly characterizes the lysosomal TRPML1 channels and its regulation in renal podocytes^77^. All these findings indicate that lysosomal Ac is an important enzyme to change sphingolipid profile in lysosomes, which may regulate TRPML1 channel activity and thereby guard against pathological fusion of lysosomes with secretory organelles such as MVBs, determining sEV secretion and osteogenesis in arterial SMCs^78^.

**Figure 10:**
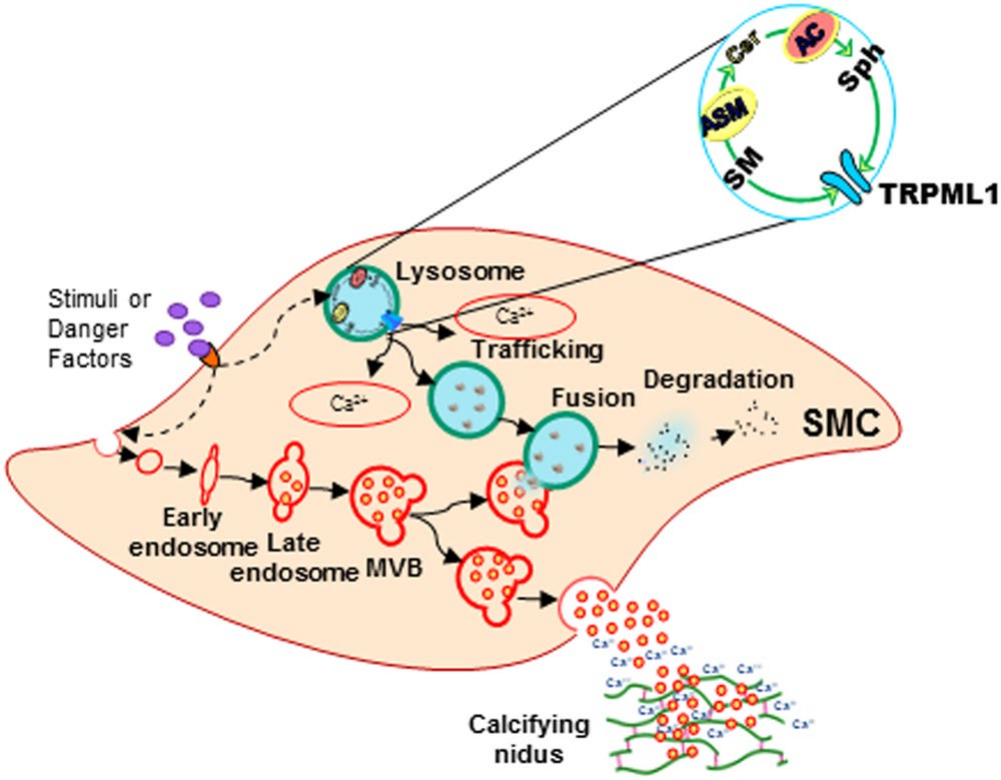
Schematic model demonstrating lysosomal Ac-mediated sEV secretion from SMCs during vascular calcification. Lysosomal Ac controlling ceramide metabolism involved in regulation of lysosomal TRPML1-mediated Ca^2+^ release and interactions of lysosome and MVBs which is associated with sEV excretion in arterial SMCs during vascular calcification. Ac: Acid ceramidase; SMCs: Smooth muscle cells.

In summary, the present study demonstrated that lysosomal Ac controlling CER metabolism may be crucially involved in phenotype maintenance and sEV excretion in arterial SMCs, which is associated with its action on the regulation of lysosomal TRPML1-mediated Ca^2+^ release and interactions of lysosome and MVBs as shown in Fig. 10. Deletion of lysosomal *Asah1* gene specifically in SMCs resulted enhanced sEV secretion, SMC phenotypic transition and AMC. Failure of TRPML1 channel activation in arterial SMCs due to Ac deficiency may lead to the reduction of lysosome-MVB interaction and consequent prolongation of MVBs fate resulting in enhanced SMCs-derived sEV release initiating AMC. These findings provide novel insights into the molecular mechanisms mediating AMC, and probably indicate new therapeutic targets for the prevention and treatment of AMC.

## Material and Methods

### Reagents and antibodies

Cholecalciferol (vitamin D3) (C9756, Sigma Aldrich, St. Louis, MO, USA). Mouse monoclonal antibody against α-SMA (ab7817, Abcam, USA), rabbit polyclonal antibodies against acid ceramidase-α (AC^α^) (sc-292176, Santa cruz, USA), RUNX2 (ab23981, Abcam, USA), OSP (ab63856, Abcam, USA), SM22-α (ab14106, Abcam, USA), VPS16 (Cat. No.17776-1-AP, Protein biotech group, USA), Rab7 (ab137029, Abcam, USA), CD63 (ab216130, Abcam, USA), annexin-II (AnX2, ab41803, USA), and alkaline phosphatase (ALP, sc-28904, Santa Cruz, USA). Rat monoclonal anti-mouse Lamp-1 (ab25245, Abcam, USA), Cre (Cat No. 6905, Novagen EMD Millipore, Billerica MA, USA), and ceramide (MID 15B4, Enzo ALX-804-196-T050). Secondary antibodies are Alexa-488 or Alexa-555-labeled (Life technologies, USA). Von Kossa staining kit (ab150687, Abcam, USA) and Alizarin Red S Solution (TMS-008-C, EMD Millipore. USA) were used for detection AMC.

### Primary culture of mouse CASMCs and alizarin red s staining

Mouse CASMCs were isolated as described previously^79^. CASMCs were cultured in Dulbecco’s modified Eagle’s medium (DMEM,Gibco), supplemented by 10% FBS (Gibco) and 1% penicillin-streptomycin (Gibco) in humidified 100% air and 5% CO_2_ mixture at 37 °C. 1 × 10^5^ cells were prepared in 6-well plates overnight, 80–90% confluent cells were treated with or without high phosphate (P_i_) (3 mmol/l)^80^, sEV release inhibitor-GW5869 (5 μM)^81^ and then incubated for 24 hours various molecular biology assays and 2 weeks for *in vitro* calcification model^82^. PBS was used to rinse CASMCs, incubated for 10 min with 4% paraformaldehyde (PFA) followed by washing three times with DH_2_O. Then Alizarin Red S staining solution was used to stain these cells for 5 min to observe calcium deposition followed by washing with DH_2_O. Finally cells were imaged under a phase microscope^83^.

### Western blot analysis

Briefly, equal amount of protein was loaded into the wells and resolved on SDS-PAGE gels along with the molecular weight marker which is then transferred to PVDF membrane. The membrane was blocked, followed by incubation with primary antibodies rabbit polyclonal to SM22-α (1:10000, abcam, USA), Rabbit polyclonal to Osteopontin (1:1000, abcam, USA), Rabbit polyclonal to RUNX2 (1:1000, abcam, USA) and rabbit anti-β-actin (1:10000, Santa Cruz Biotechnology, Dallas, TX, USA) overnight at 4 °C followed by incubation with donkey anti-rabbit-HRP IgG (1:5000, Santa Cruz Biotechnology, Dallas, TX, USA) for 1 hour at room temperature. Finally, bands were detected by chemiluminescence technique using LI-COR Odyssey Fc and the band intensity of target proteins were normalized to β-actin and calculated with Image J software version 1.44p (NIH, Bethesda, MD, USA)^84^.

### Real time-PCR studies

RT-PCR was used to quantify the mRNA expression of SM22-α, Osteopontin and RUNX2 in the wild type and *Asah1*^fl/fl^SM^Cre^ CASMCs^83^. The following mouse specific primers were used: β-actin, sense 5′-TCGCTGCGCTGGTCGTC-3′ and antisense 5′-GGCCTCGTCACCCACATAGGA-3′; SM22-α, sense 5′-TCCAGTCCACAAACGACCAAGC-3′ and antisense 5′-GAATTGAGCCACCTGTTCCATCTG-3′; OSP, sense 5′-ATTTGCTTTTGCCTGTTTGG-3′ and antisense 5′-CTCCATCGTCATCATCATCG–3′; RUNX2 sense 5′-CCAGATGGGACTGTGGTTACC-3′ and antisense 5′-ACTTGGTGCAGAGTTCAGGG-3′. To normalize mRNA expression, β-Actin was used as internal control. Fold changes were determined as follows: 2^−ΔΔ threshold cycle^ ^84^.

### Nucleofection

CASMCs were transfected with GCaMP3-ML1 plasmid via nucleofector technology developed by Lonza (Basel, Switzerland). This technology use electrical impulses to generate temporary small pores in the membrane together with cell-specific solutions which deliver substrates through the cytoplasm and into the nuclear membrane. CASMCs (1 × 10^6^ cells) were gently centrifuged (90xg, 10 min, RT) and resuspended in SF Cell Line nucleofector solution containing 2 μg plasmid DNA. Then the whole cell suspension was transferred into a certified cuvette and subjected to cell-type specific program DS-150 in Nucleofector system, chosen based on optimization experiments. Finally, pre-warmed medium was used to resuspend the Nucleofected cells and transferred to cultured plates for use in experiments^77^.

### Ca^2+^ imaging

Post-nucleofection (18–24 hrs) with GCaMP3-ML1, CASMCs were ready for further experiments. The fluorescence intensity was recorded at 470 nm (F470) with a digital camera (Nikon Diaphoto TMD Inverted Microscope). Metafluor imaging and analysis software were used for offline processing of images and statistical analysis (Universal Imaging, Bedford Hills, NY, USA). Low external Ca^2+^ solution

(145 mM NaCl, 5 mM KCl, 3 mM MgCl2, 10 mM glucose, 1 mM EGTA and 20 mM HEPES (pH 7.4) was used to measure lysosomal Ca^2+^ release^77^.

### Whole-lysosome patch clamp recording

Size of the lysosomes of CASMCs were increased with vacuolin-1 (1 µM) for 2 hours. Isolated lysosomes from CASMCs were used for the whole-lysosome electrophysiology using a planar patch clamp system, Port-a-Patch (Nanion Technologies)^85^. Prior to the measurements, bath solution containing 60 mM KF, 60 mM KMSA, and 10 mM HEPES (pH 7.2, 2 mM CaMSA) was added immediately to avoid precipitation of CaF_2_. The luminal solution contained 60 mM CaMSA, 70 mM KMSA, 2 mM MgCl_2_, and 10 mM HEPES (pH 4.6). The planar patch-clamp technology combined with a pressure control system and microstructured glass chips containing an aperture of around 1μm diameter (resistances of 10–15 MΩ) (Nanion Technologies). Currents were recorded using an EPC-10 patch-clamp amplifier and Patch Master acquisition software (HEKA). Data were digitized at 40 kHz and filtered at 2.8 kHz. The membrane potential was held at −60mV, and 500 ms voltage ramps from −200 to +100 mV were applied every 5 s. All recordings were took at room temperature (21–23 °C), and analyzed using Patch Master (HEKA) and Origin 6.1 (OriginLab)^77^.

### Isolation of small extracellular vesicles (sEVs)

Using differential ultracentrifugation technique, sEVs were extracted from CASMCs culture medium as mentioned earlier^18^. As described above, confluent CASMCs cells were treated with vehicle or P_i_ (3 mmol/l)^80^ for 24 hrs. To eliminate detached cells, cell medium was collected in centrifuge tubes and centrifuged at 300 g at 4 °C for 10 min. Supernatant was collected and purified by filtering through 0.22 µm filters to eliminate contaminating apoptotic bodies, microvesicles and cell debris. The filtered supernatant was then ultracentrifuged at 100,000 × g for 90 min at 4 °C (Beckman 70.1 T1 ultracentrifuge rotator) to obtain sEVs pellet. Finally, pellet was washed with the PBS at 100,000 × g and resuspended in 50 μl of ice-cold PBS. PBS was used for further dilutions during nanoparticle analysis^83^.

### Nanoparticle tracking analysis (NTA)

Light scattering mode of the NanoSight LM10 (Nano Sight Ltd., Amesbury, United Kingdom) was used to analyze the CASMC-derived sEVs during nanoparticle tracking analysis (NTA)^86^. Samples were diluted in PBS and 5 frames (30 s each) were captured for each sample with background level 10, camera level 12 and shutter speed 30. Captured video was analyzed using NTA software (Version 3.2 Build 16) and an average size distribution graph was plotted using PRISM software (GraphPad, San Diego, CA)^18^.

### Vitamin D-mediated AMC mouse model

SM-specific *Asah1* gene knockout (*Asah1*^fl/fl^/SM^Cre^) mice (N-AcylsphingosineAmidohydrolase 1 (*Asah1*) were used in the present study. 12–14-weeks-old male C57BL/6 J wild-type and *Asah1*^fl/fl^/SM^Cre^ mice were used. Mice were characterized by genotyping, *in vivo*/*ex-vivo* imaging and confocal microscopy. All protocols were endorsed by the Virginia Commonwealth University Institutional Animal Care and Use Committee, and all experiments performed in accordance with relevant guidelines and regulations. Animals were subsequently randomized into 6 groups, for each mouse strain (*Asah1*^fl/fl^/SM^Cre^, *Asah1*^fl/fl^/SM^wt^ and WT/WT) subcutaneously injected active vitamin D (Vit D) (500,000 IU/Kg/bw/day) or matched vehicle (5% v/v ethanol) for 3–4 days^83^. After 16–17 days of post injection period, animals were anaesthetized with 2% isoflurane through a nose cone. Blood was withdrawn and plasma was isolated, stored at −80 °C. Mice were sacrificed, heart and aorta were collected, with a section of each stored in 10% buffered formalin for histopathological examinations and immunostaining. And the remaining section of heart and aorta were frozen using liquid nitrogen, and stored at −80 °C for frozen tissue slides to perform dual fluorescence staining and confocal microscopy.

In mouse model of AMC high doses of Vit D were used to generate the AMC mouse model^87^. High doses of Vit D (500,000 IU/Kg/bw/day) were subcutaneously injected to normal C57BL/6 N, *Asah1*^fl/fl^/SM^wt^ and *Asah1*^fl/fl^/SM^Cre^ mice for 3–4 days (n = 5–6 per group). The Vit D solution was formulated as follows: Absolute ethanol (200 μl) was used to dissolve vitamin D3 (66 mg) and mixed with 1.4 ml of cremophor (Sigma Aldrich) and left at RT for 15 minutes. After that dextrose (750 mg) dissolved in sterilized water (18.4 ml) was added to Vit D solution and left RT for 15 minutes. The prepared Vit D solution was stored at 4 °C until used, but generally made fresh after couple of days^83^.

### Alizarin Red S staining, Von Kossa staining and immunohistochemical analyses

Post 16–17 days of Vit D injection, mice were anaesthetized using 2% isoflurane through a nose cone. The mouse heart and aorta dissected from the mice were fixed in 10% buffered formalin for 24 hrs and then embedded in paraffin to make tissue slides. For AMC detection, Alizarin Red S staining was performed^83^. Deparaffinization of samples were carried through different washes of alcohol and xylene followed by distilled water. 1% Alizarin Red S solution was used to stain the arterial sections for 5 min followed by 20 sec incubation with acetone and another 20 sec with acetone-xylene. The reddish color indicates positively stained. Von Kossa staining was carried out to observe mineralization as described earlier^88^. Briefly, deparaffinized samples were rinsed with Milli-Q water three times. Samples were then treated with 1% aqueous silver nitrate solution and incubated under a UV lamp for 1 hour or more followed by washing three times with Milli-Q water at RT. Incubation with 2.5% sodium thiosulfate for 5 minutes eliminated non-specific staining. Nuclear staining was carried out by incubating samples with nuclear red fast stain for 5 minutes. Finally, samples were dehydrated and mounted.

Paraffin sections were made using fixed mouse aortas for histological examinations, where tissues were dehydrated and embedded in paraffin and cut into 5 μm sections. Immunohistochemical analyses were conducted as mentioned previously^89^ or following the manufacturer’s protocol for CHEMICON IHC Select HRP/DAB Kit (EMD Millipore, MA)^83^. Briefly, after antigen retrieval using citrate buffer, 3% H_2_O_2_ was used to quench endogenous peroxidase activity. 2.5% horse serum was used to block the aortic tissue for 1 hour at RT followed by incubation with primary antibodies against RUNX2 (1:300), OSP (1:100), CD63 (1:50), AnX2 (1:50) and ALP (1:50) overnight at 4 °C and afterwards with biotinylated secondary antibodies and a streptavidin peroxidase complex (PK-7800, Vector Laboratories, Burlingame, CA, USA). The aortic tissue slides were treated sequentially in compliance with manufacturer’s protocol. Aortic tissue were eventually counterstained with hematoxylin, dehydrated and mounted using DPX. Finally, Image Pro Plus 6.0 software was used to measure the area percentage of the positive staining^83,90^.

### Immunofluorescence staining

Indirect immunofluorescent staining was performed to evaluate co-localization of the MVB protein marker VPS16 against lysosomal protein marker Lamp-1, representing MVB-lysosome interaction in SMCs. Acetone fixed frozen aortic tissue slides were incubated with indicated primary antibodies VPS16 (1:300) and (Lamp-1) (1:200) at 4 °C overnight. Double immunofluorescent staining was accomplished by incubating slides with Alexa-488 or Alexa-555-labeled secondary antibodies for 1 hour at RT. Finally, DAPI-mounting solution was used to mount the slides, and then examined using confocal laser scanning microscope (Fluoview FV1000, Olympus, Japan). As mentioned earlier^90,91^ images were examined by the Image Pro Plus 6.0 software (Media Cybernetics, Bethesda, MD), which measured and represented co-localization as the Pearson Correlation Coefficient (PCC)^83^.

### X-gal staining

X-gal staining was performed as per the manufacturer’s instruction (Beta Blue Staining Kit, Novagen catalog #71074-3).

### Calcium and phosphate assay

Plasma calcium and phosphate levels were estimated by kits available commercially, (ab102505, Abcam, USA) and (ab65622, Abcam, USA) following manufacturer’s protocol.

### Statistical analysis

All of the values are expressed as mean ± SEM. Significant differences among multiple groups were examined using two-way ANOVA followed by a Duncan’s test. P < 0.05 was considered statistically significant.

## Supplementary information

Supplementary information.
