# Supplementary material for: Arterial Medial Calcification through Enhanced small Extracellular Vesicle Release in Smooth Muscle-Specific Asah1 Gene Knockout Mice
Source: Sci Rep. 2020 Feb 3;10:1645. doi: 10.1038/s41598-020-58568-5 (PMC6997457; doi:10.1038/s41598-020-58568-5)
Supplement: Supplementary file 1 — Supplementary information. [file 41598_2020_58568_MOESM1_ESM.pdf]

Arterial Medial Calcification through Enhanced small Extracellular Vesicle Release in Smooth Muscle-Specific *Asah1* Gene Knockout Mice

Owais M. Bhat[1], Guangbi Li[1], Xinxu Yuan[1], Dandan Huang[1], Erich Gulbins[2], Rakesh C. Kukreja[3], Pin-Lan Li[1]

[1]Department of Pharmacology and Toxicology, Virginia Commonwealth University, School of Medicine, Richmond, VA 23298

[2]Department of Molecular Biology, University of Duisburg-Essen, Essen, Germany and Dept. of Surgery, University of Cincinnati, Cincinnati, USA.

[3]VCU Pauley Heart Center, Division of Cardiology, Virginia Commonwealth University, 1101 East Marshall Street, Richmond, VA 23298-0204

Running title: Arterial medial calcification in SM-specific *Asah1* KO mice

Correspondence should be addressed to:

Pin-Lan Li, M.D, Ph.D
Department of Pharmacology & Toxicology
Medical College of Virginia Campus
Virginia Commonwealth University
Richmond, VA 23298
Tel: (804) 828-4793
Fax: (804) 828-4794
E-mail: pin-lan.li@vcuhealth.org

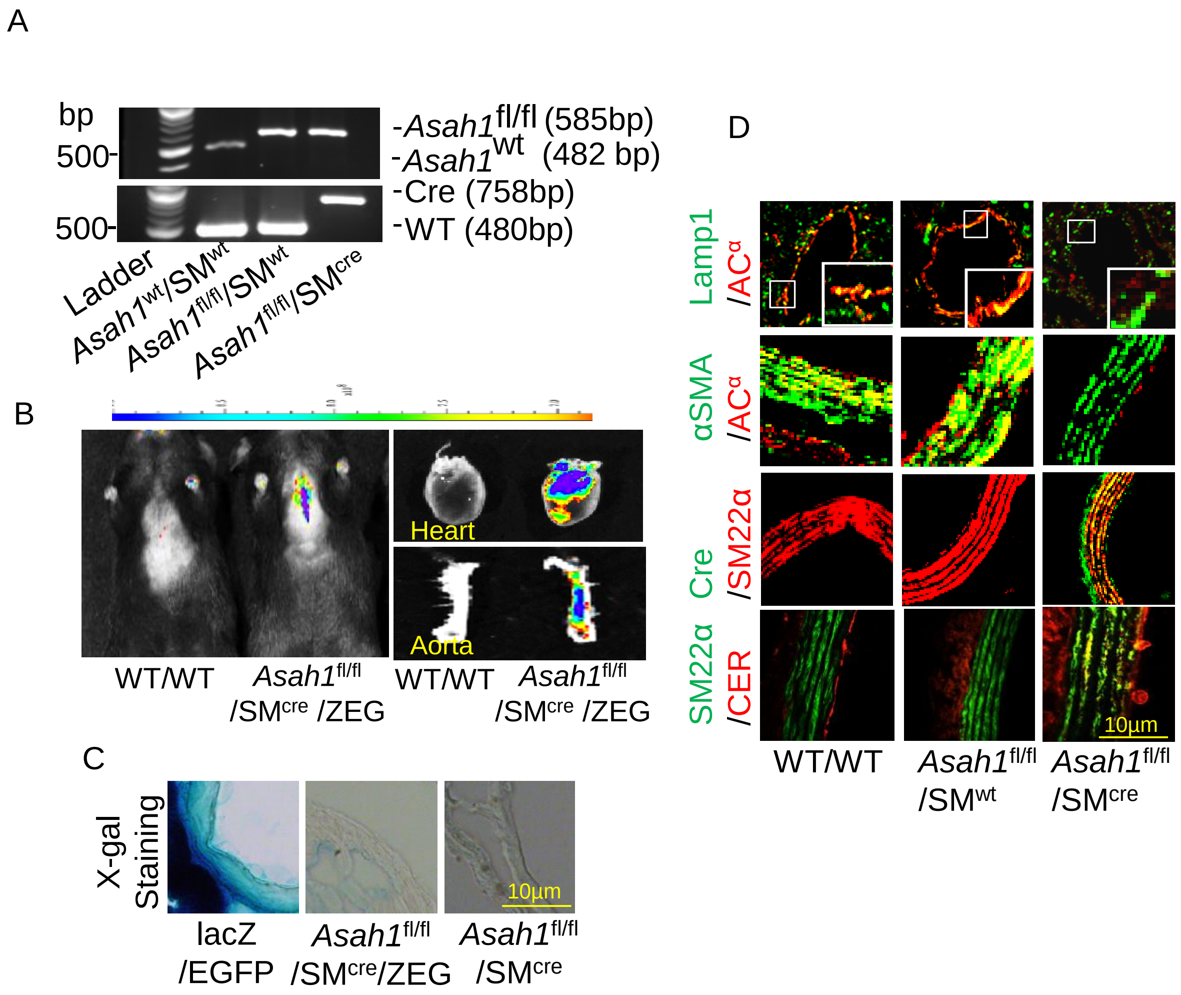

Supplementary Fig. S1 Characterization of SM-specific *Asah1* knockout (KO) mice. **A.** *Asah1*$^{fl/fl}$/SM^{Cre} has 2 positive PCR products including 758 bp for Cre and 585 bp for floxed *Asah1* gene. Asah*1*$^{fl/fl}$/SM^{wt} mice had positive floxed *Asah1* gene (585 bp), but no Cre (758 bp). Deletion of the *Asah1* was verified by PCR analysis. **B.** Cre-mediated SM-specific recombination was validated by breeding the *Asah1*$^{fl/fl}$/SM^{Cre} mice with Z/EG reporter mice, *in vivo* and *ex vivo* imaging of the offspring showed GFP expression in the aorta and coronary arteries of heart. **C.** Representative X-gal staining in the coronary arterial wall of mice. **D.** Representative fluorescent confocal microscopic images showed no co-localization of AC-α protein (red) with Lamp-1 (green, as a lysosome protein marker) in coronary arterial wall or α-SMA (green, as SMC marker) in aortic wall was observed in *Asah1*$^{fl/fl}$/SM^{Cre} compared to their littermates (*Asah1*$^{fl/fl}$/SM^{wt} and WT/WT). In contrast, Cre was only detected in aortic SMCs of *Asah1*$^{fl/fl}$/SM^{Cre} as shown in colocalization of Cre (green) vs. SM22-α (red, SMC marker). CER was markedly increased SMCs of *Asah1*$^{fl/fl}$/SM^{Cre} as shown in colocalization of CER (red) vs. SM22-α (green, SMC marker) . Yellow spots or patches in the overlaid images were defined as colocalization of AC-α, CER & Cre with smooth muscle cell markers and Lamp-1 in arterial wall. SM (smooth muscle cell): AC-α: acid ceramidase-α, CER: ceramide, Cre: cre recombinase protein, α-SMA: α-smooth muscle cell actin (smooth muscle cell marker), Lamp-1: lysosomal associated membrane protein-1: SM22-α: smooth muscle cell marker: SM (smooth muscle cell). (n=3)

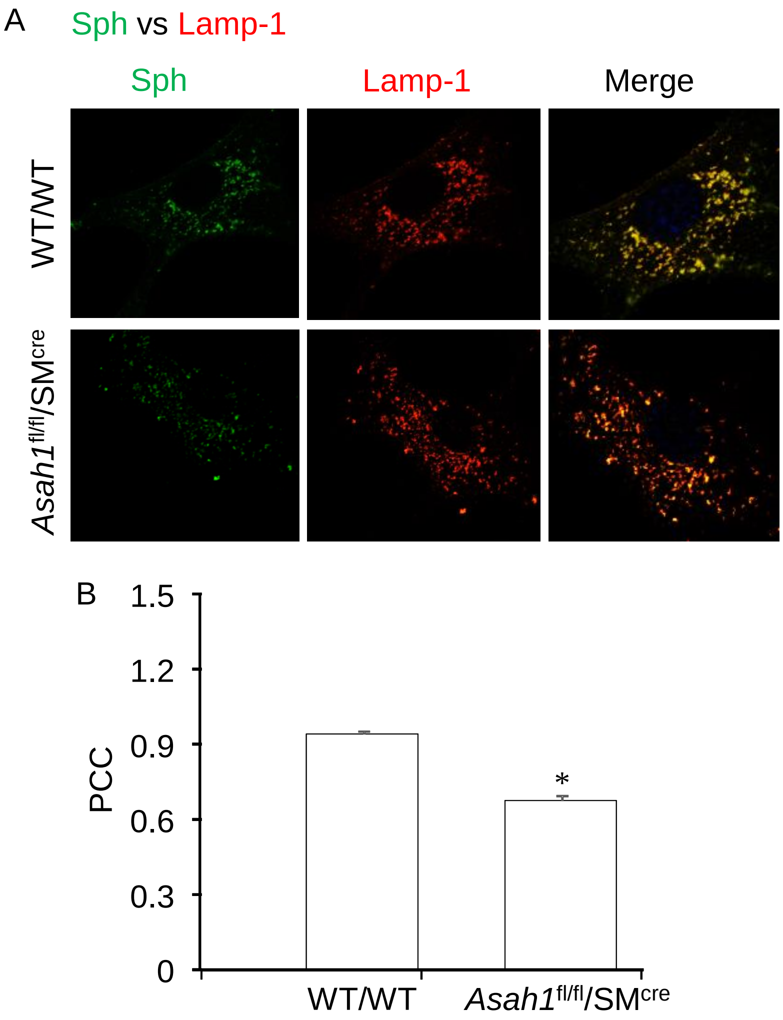

Supplementary Fig. S2 **A.** Representative confocal images showed colocalization of Sph (green) and Lamp-1(red), and also sphingosine staining in *Asah1*$^{fl/fl}$/SM^{Cre} CASMCs was much lower as compared to WT type cells **B.** Bar graph shows Ac gene deletion significantly decreased colocalization of Sph/Lamp-1 in *Asah1*$^{fl/fl}$/SM^{Cre} CASMCs, n=3. * $P < 0.05$ vs. WT/WT group. Lamp-1: lysosomal associated membrane protein-1: Sph: Sphingosine.

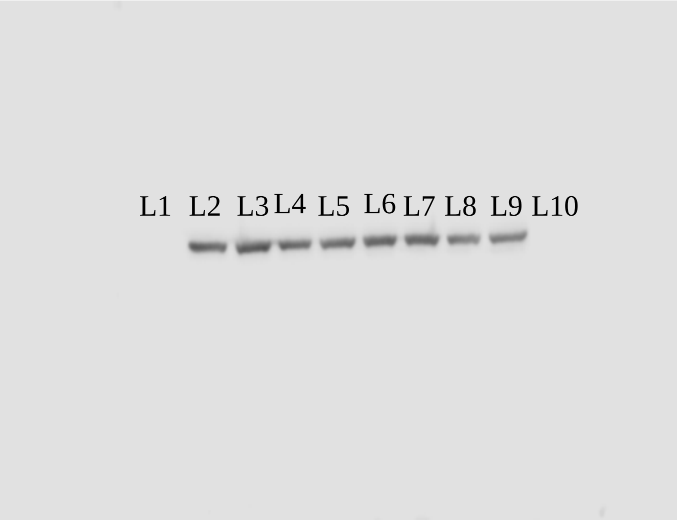

Original blot of beta-actin (OSP) Fig 5

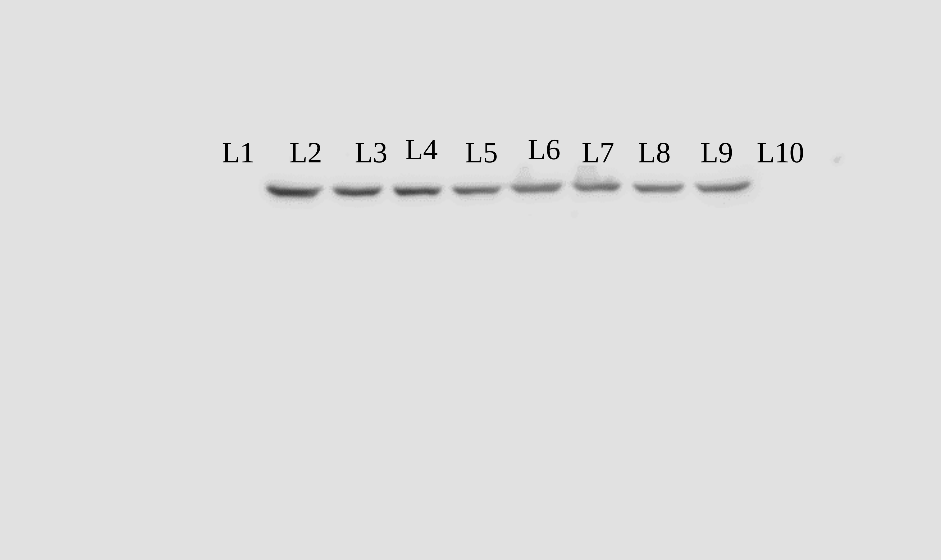

L1-Ladder
L2-WT Vehl
L3-WT Pi
L4-Asah$^{fl/fl}$SM^{cre} Vehl
L5-Asah$^{fl/fl}$SM^{cre} P_i
L6-WTVehl
L7-WTP_i
L8-Asah$^{fl/fl}$SM^{cre} Vehl
L9-Asah$^{fl/fl}$SM^{cre} P_i
L10-Ladder

Original blot of beta-actin (RUNX2) Fig 5

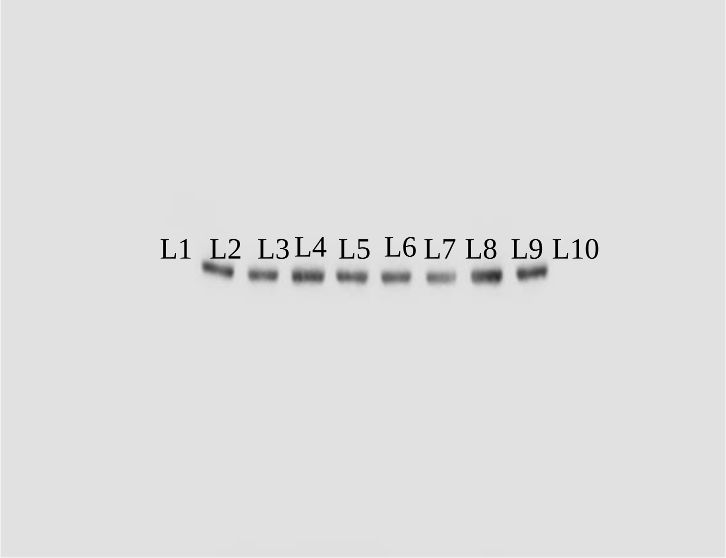

Original blot of beta-actin (SM22 alpha) Fig 5

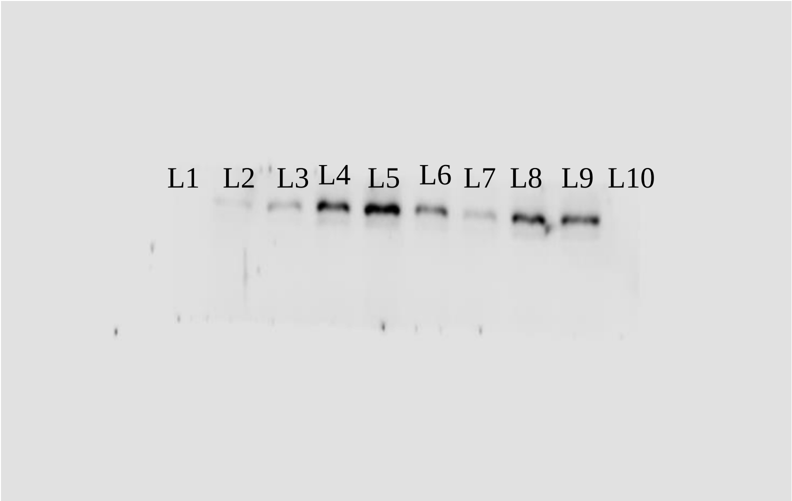

Original blot of OSP Fig 5

L1-Ladder
L2-WT Vehl
L3-WT Pi
L4-Asah$^{fl/fl}$SM^{cre} Vehl
L5-Asah$^{fl/fl}$SM^{cre} P_i
L6-WTVehl
L7-WTP_i
L8-Asah$^{fl/fl}$SM^{cre} Vehl
L9-Asah$^{fl/fl}$SM^{cre} P_i
L10-Ladder

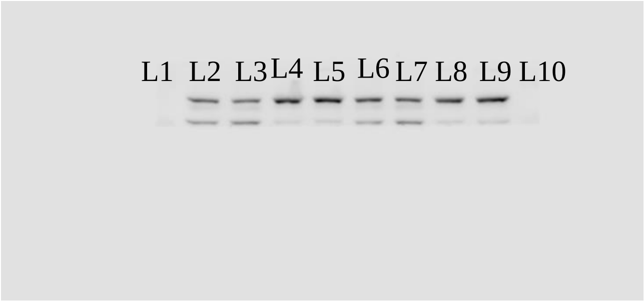

Original blot of RUNX2 Fig 5

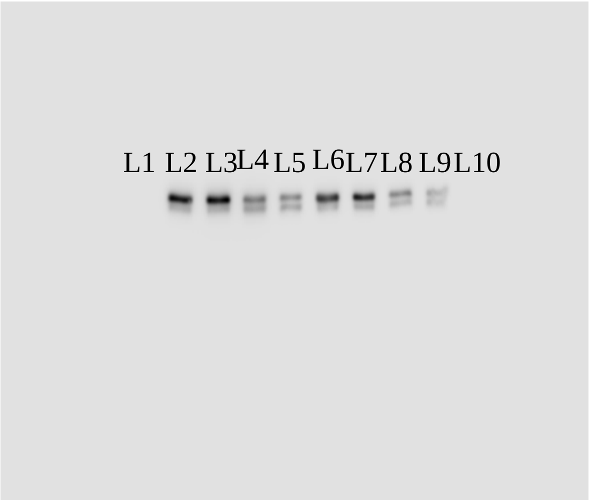

Original blot of SM22 alpha Fig 5
